# Loss of transcription factor EB dysregulates the G1/S transition and DNA replication in mammary epithelial cells

**DOI:** 10.1016/j.jbc.2022.102692

**Published:** 2022-11-11

**Authors:** Logan Slade, Dipsikha Biswas, Petra C. Kienesberger, Thomas Pulinilkunnil

**Affiliations:** Department of Biochemistry and Molecular Biology, Dalhousie University, Dalhousie Medicine New Brunswick, Saint John, New Brunswick, Canada

**Keywords:** cell cycle, transcription factor, TFEB, DNA replication, origin licensing, triple negative breast cancer, DNA damage, genome stability, RNA-Seq, Aurora kinase A, Stathmin 1, RB1, MDA-MB-231, MCF-10A, AURKA, Aurora kinase A, CLEAR, coordinated lysosomal expression and regulation, dT, thymidine, HU, hydroxyurea, HOMER, Hypergeometric Optimization of Motif EnRichment, PhPy, phthalazinone pyrazole, TNBC, triple-negative breast cancer, TFEB, transcription factor EB

## Abstract

Triple-negative breast cancer (TNBC) poses significant challenges for treatment given the lack of targeted therapies and increased probability of relapse. It is pertinent to identify vulnerabilities in TNBC and develop newer treatments. Our prior research demonstrated that transcription factor EB (TFEB) is necessary for TNBC survival by regulating DNA repair, apoptosis signaling, and the cell cycle. However, specific mechanisms by which TFEB targets DNA repair and cell cycle pathways are unclear, and whether these effects dictate TNBC survival is yet to be determined. Here, we show that TFEB knockdown decreased the expression of genes and proteins involved in DNA replication and cell cycle progression in MDA-MB-231 TNBC cells. DNA replication was decreased in cells lacking TFEB, as measured by EdU incorporation. TFEB silencing in MDA-MB-231 and noncancerous MCF10A cells impaired progression through the S-phase following G1/S synchronization; however, this proliferation defect could not be rescued by co-knockdown of suppressor RB1. Instead, TFEB knockdown reduced origin licensing in G1 and early S-phase MDA-MB-231 cells. TFEB silencing was associated with replication stress in MCF10A but not in TNBC cells. Lastly, we identified that TFEB knockdown renders TNBC cells more sensitive to inhibitors of Aurora Kinase A, a protein facilitating mitosis. Thus, inhibition of TFEB impairs cell cycle progress by decreasing origin licensing, leading to delayed entry into the S-phase, while rendering TNBC cells sensitive to Aurora kinase A inhibitors and decreasing cell viability. In contrast, TFEB silencing in noncancerous cells is associated with replication stress and leads to G1/S arrest.

Breast cancer is the most frequently diagnosed cancer among women and is a leading cause of cancer-related death (1). Molecular heterogeneity among breast tumors results in differing patient outcomes according to disease subtypes (2, 3). Triple-negative breast cancer (TNBC) represents 10 to 15% of all breast cancer diagnoses and is defined by the lack of estrogen receptor, progesterone receptor, and HER2 expression (2). TNBC has the worst prognosis of all breast cancer subtypes, with a 4-year survival rate of 77% (3). Targeted therapies have not been developed for the treatment of TNBC; therefore, cytotoxic chemotherapies are the standard of care, typically a combination of taxanes and anthracyclines (4, 5, 6). TNBC is responsive to systemic chemotherapy due to the highly proliferative nature of the subtype; however, subgroupings of TNBC have been identified that are more resistant to these treatments (7, 8). Despite the increased response rates compared with non-TNBC patients, the long-term rates of progression-free and overall survival are considerably lower for TNBC. 76% of non-TNBC patients have progression-free survival for 3 years compared to 63% of TNBC patients (9). Indeed, the risk of distant recurrence within 5 years is ∼2.5 times greater for TNBC than non-TNBC. Hence, TNBCs are partially sensitive to cytotoxic chemotherapy, but only one-third of patients completely respond. Furthermore, residual disease, recurrence, and treatment resistance is an outstanding clinical challenge that drives worse outcomes for TNBC. These factors compel greater investigation into the molecular mechanisms that sustain the growth of TNBC.

Our prior research has found that transcription factor EB (TFEB) is an essential protein for the growth and proliferation of TNBC cells (10). TFEB is part of the MiT/TFE family of transcription factors, including MITF, TFE3, and TFEC (11). TFEB activates the transcription of genes in proximity to the six base pair E-Box promoter and the related eight base pair CLEAR (coordinated lysosomal expression and regulation) promoter (12, 13, 14). TFEB has previously been identified as the master transcriptional regulator of autophagy-lysosome gene expression, given that many of the genes critical for the biogenesis and function of lysosomes possess CLEAR sequences (13, 14). Surprisingly, our prior research found that lysosomal function was dispensable for the prosurvival activity of TFEB in TNBC cells. Instead, we found that TFEB regulated DNA damage repair, apoptosis, and cell cycle gene expression (10).

Constitutive activation of the cell cycle is necessary for the growth of all cancers, including breast cancer (15, 16). In TNBC, elevated gene expression of cell cycle genes is characteristic of the subtype and correlates with increased markers of cell proliferation in patients (17, 18). Treatments that inhibit the cell cycle are regularly used as breast cancer chemotherapy (19). For ER+ breast cancer, CDK4/6 inhibitors have proven effective; although, their utility is limited in TNBC patients due to frequent deletions of RB1 and copy number amplifications of cyclin E1, which allows bypass of the G1/S checkpoint (16, 20, 21). A consequence of elevated cell proliferation in TNBC is replication stress, which arises from the impaired progression of DNA replication machinery due to DNA lesions, DNA secondary structures, conflicts with transcription, and nucleotide shortages (22). The consequences of replication stress include DNA double strand breaks and genomic instability (22). TNBC tumors exhibit high levels of replication stress, which is associated with higher levels of cyclin E and deletion of PTEN, while replication stress correlates with sensitivity to the immune checkpoint and PARP inhibition (23, 24, 25). Therefore, dysregulated cell cycle progression and replication stress are promising therapeutic targets in TNBC.

Transcriptomics analysis showed that TFEB knockdown in MDA-MB-231 TNBC cells resulted in global downregulation of cell cycle genes in conjunction with diminished DNA repair capacity and increased apoptosis (10). Given that TNBC is characterized by genetic upregulation of the cell cycle, we questioned whether TFEB was critical for the progression of the cell cycle in TNBC. Prior studies have found that TFEB regulates G1/S progression in endothelial cells and directly regulates CDK4 gene expression in MEFs (26, 27). Currently, it is unknown whether regulation of the cell cycle by TFEB has a functional consequence in TNBC.

We hypothesized that the proliferation of TNBC cells is sustained by TFEB-dependent regulation of cell cycle and DNA replication proteins. Here, we show that TFEB silencing dysregulates the expression of cell cycle machinery at both the gene and protein level in MDA-MB-231 TNBC cells and MCF10A noncancerous breast epithelial cells. TFEB knockdown reduces cell proliferation and the number of cells in the S-phase while preventing S-phase entry following G1/S synchronization by thymidine block. Delayed S-phase entry following TFEB knockdown is not the result of elevated G1/S checkpoint signaling, rather we show TFEB silencing results in replication origin under-licensing. Lastly, we find that the reprogramming of cell cycle regulatory networks caused by TFEB knockdown sensitizes TNBC cells to Aurora kinase A (AURKA) inhibition. Our study demonstrates that regulation of the cell cycle by TFEB is necessary for TNBC cell growth.

## Results

### TFEB expression is increased in TNBC

**Figure 1:**
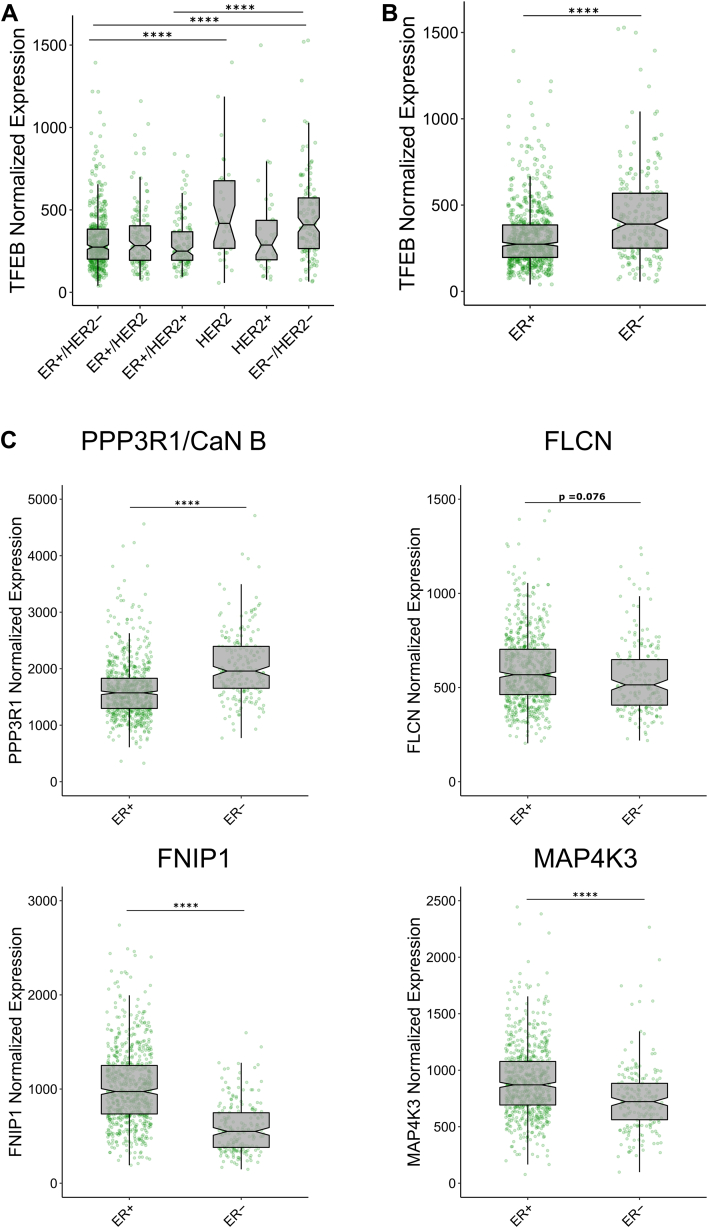
**TFEB gene expression is elevated in TNBC patients.***A* and *B*, boxplots of TFEB RSEM normalized gene expression values as measured by RNA-Seq from breast tumor biopsies collected by the TCGA: breast cancer study, separated by either IHC subtype, or IHC estrogen receptor status. *C*, *boxplots* for PPP3R1, FLCN, FNIP1, and MAP4K3 normalized expression values from breast cancer patient tumor biopsies collected as part of the TCGA: breast cancer study, delineated by estrogen receptor status. Notches on boxplots indicate Tukey confidence intervals. ∗∗∗∗*p* < 0.0001, (*A*) one-way ANOVA, or (*B* and *C*) *t* test. TCGA, The Cancer Genome Atlas; TFEB, transcription factor EB; TNBC, triple-negative breast cancer.

Our prior results found that TFEB was crucial for the survival of TNBC cells (10); however, limited studies have explored whether TFEB expression varies by molecular subtype in breast cancer patients. Gene expression data were obtained from The Cancer Genome Atlas breast cancer patient cohort, and TFEB expression was examined by histological and molecular subtypes. TFEB gene expression is significantly higher in patients with ER-/HER2-breast cancer compared to patient samples that are ER+, HER2+, or ER+/HER+ (Fig. 1, *A* and *B*). Similarly, ER status alone is associated with differing TFEB expression levels as ER− patients show significantly higher expression of TFEB than those patients with ER+ breast cancer (Fig. 1, *A* and *B*). Cox proportional hazards regression indicates that higher TFEB expression is associated with worse survival, with the hazard ratio for TFEB expression being 1.151 (95% confidence interval: 0.7182–1.844); however, the effect is not statistically significant (*p* = 0.56).

Regulators of TFEB function also show varied expression by breast cancer molecular subtype (Fig. S1, *A* and *B*). Phosphorylation of TFEB at serine 3 by MAP4K3 and recruitment to the lysosome by RagC/D is necessary for inhibition of TFEB by mTORC1, and this inhibitory phosphorylation is reversed by the phosphatase calcineurin (28). The calcineurin regulatory subunit PPP3R1 shows elevated expression in ER− breast cancer, whereas positive regulators of Rag C/D, including folliculin complex members FLCN and FNIP1, along with MAP4K3, are decreased in ER− breast cancer patients (Fig. 1*C*). These results show that TFEB expression is elevated in TNBC patients and is consistent with an expression pattern of TFEB regulatory genes that is indicative of TFEB activation. Given that TFEB is highly expressed in TNBC patients and TNBC is characterized by genetic upregulation of the cell cycle, we questioned whether TFEB was critical for the progression of the cell cycle in TNBC.

### Loss of TFEB function dysregulates cell cycle gene expression

**Figure 2:**
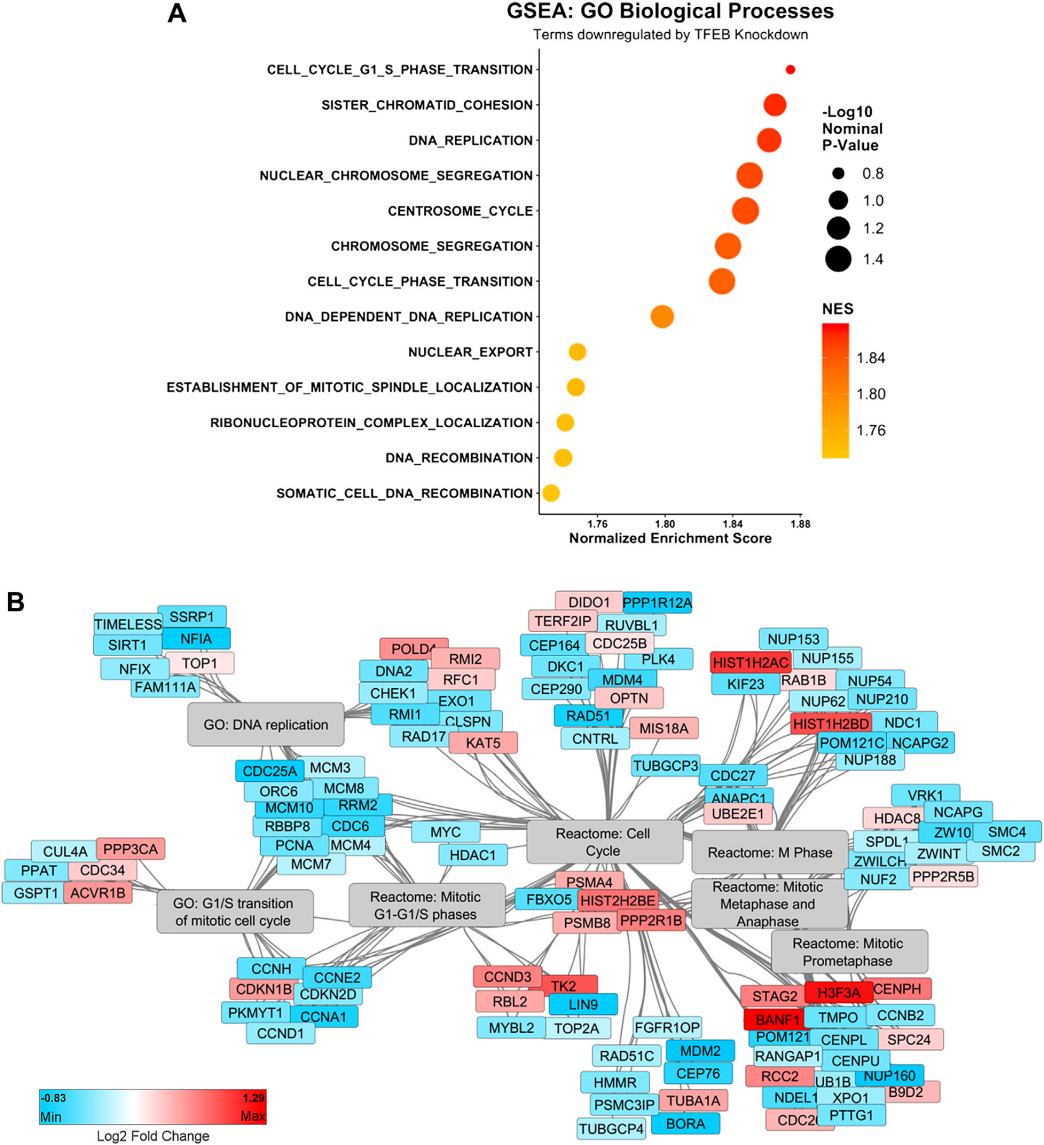
**Cell cycle genes are globally downregulated by TFEB knockdown.***A*, gene set enrichment analysis with RNA-Seq gene expression results from TFEB knockdown MDA-MB-231 cells (GSE139203), ordered by the normalized enrichment score. *B*, network analysis of significantly differentially expressed genes related to the cell cycle and associated GO and Reactome terms. TFEB, transcription factor EB.

We next examined the effect of TFEB knockdown on global gene expression in MDA-MB-231 cells utilizing our previously published RNA-Seq dataset (10). In brief, MDA-MB-231 cells were transfected with either of two siRNA’s targeting TFEB exon 4 and 7, respectively, or with the nontargeting control siRNA and cultured for 48 h before RNA was extracted and analyzed. This transcriptomic analysis of MDA-MB-231 TNBC cells showed that gene ontology terms related to cell cycle genes were enriched in the subset of genes downregulated by TFEB silencing (Fig. 2*A*). Specifically, the most significantly downregulated gene ontology terms included “Cell Cycle G1/S Phase Transition”, “Sister Chromatid Cohesion”, and “DNA Replication” (Fig. 2*A*). The significantly altered genes associated with these terms include key cell cycle regulators such as cyclin D1, cyclin E2, cyclin A1, and cyclin B2 (Fig. 2*B*). Lastly, proteins required for mitosis are decreased by TFEB knockdown, such as condensin subunits SMC2 and SMC4, together with centromere proteins CENPU and CENPL (Fig. 2*B*).

### Promoter motif enrichment identifies transcription factors dysregulated by TFEB in TNBC

Our findings show that TFEB knockdown alters a significant number of cell cycle–related genes in MDA-MB-231 cells; however, it is unclear how many of these genes are directly regulated by TFEB transcriptional activity. To identify TFEB targets that are downregulated by TFEB silencing, HOMER (Hypergeometric Optimization of Motif EnRichment) was used to identify CLEAR motifs near the transcription start site of genes significantly downregulated by both siRNAs with a log_2_ fold change of less than -0.3. This methodology identified 54 unique genes containing several CLEAR sequences (Table S2). These genes are involved in various cellular processes, including metabolism: AGPS, CAD, GK, ALDH6A1, SLC25A32, METAP1D, COA7, and DPH2. ALDH6A1 is required for amino acid oxidation, CAD is a key enzyme in pyrimidine nucleotide biosynthesis, and COA7 is part of the mitochondrial electron transport chain. Several other genes encode for proteins involved in RNA processing, including RNA splicing factors (ESRP2, HNRNPA3) and ribosomal RNA or tRNA biosynthesis components (TSEN2, RRP9, UTP20, and ESF1). Few of the genes that were downregulated by TFEB silencing and directly involved in the cell cycle contained the canonical CLEAR sequence. Two of the identified genes, ZNF207/BuGZ and WDR62, are involved in spindle assembly and chromosomal separation during mitosis (29, 30). ZNF207 activates the mitotic kinase: AURKA, while WDR62 is a downstream target of AURKA (31, 32, 33).

**Figure 3:**
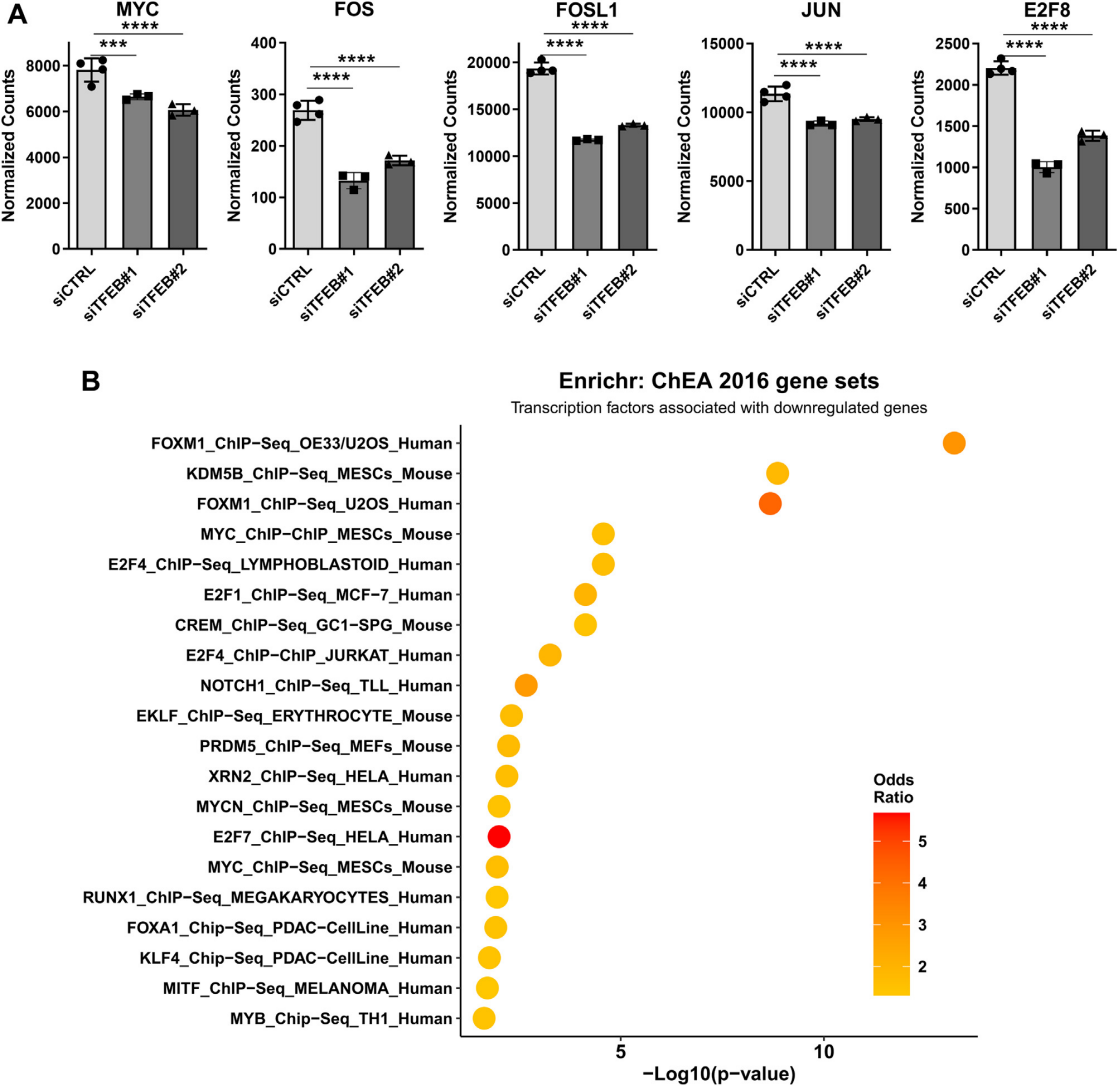
**Transcriptional activators of cell growth are downregulated by TFEB knockdown.***A*, gene expression of the indicated transcription factors as determined by RNA-Seq analysis of MDA-MB-231 cells with or without knockdown of TFEB, presented as DESeq2 normalized counts. *B*, genes significantly downregulated by TFEB knockdown were subjected to enrichment analysis against a database of ChIP-Seq results (ChEA) using Enrichr, and the significantly enriched chromatin factors displayed ordered by -log10 *p*-value of enrichment, with the color representing the ratio of enrichment. ∗∗∗*p* < 0.001, ∗∗∗∗*p* < 0.0001. TFEB, transcription factor EB.

Given that much of the differential gene expression resulting from TFEB silencing is not due to the canonical action of TFEB, we next considered if any other transcription factor networks could be altered by loss of TFEB function. To study this question, the promoter region of genes significantly downregulated by both TFEB siRNAs with a log_2_ fold change of less than -0.3 were subjected to “known” motif enrichment analysis using HOMER. This method identified several enriched promoter motifs, with the two most significant being motifs for nuclear transcription factor Y family and Basic Leucine Zipper ATF-Like Transcription Factor (Table 1). Other notable motifs with enrichment include those for AP-1/FOS, cMYC, and E2F7/8 (Table 1). Reflecting the decrease in expression of transcription factor networks, RNA-Seq results show that gene expression for AP-1 components FOS, JUN, and FOSL1 are significantly decreased by knockdown of TFEB in MDA-MB-231 cells (Fig. 3*A*). Likewise, MYC and E2F8 showed significantly decreased expression in TFEB silenced cells as measured by RNA-Seq transcriptomics (Fig. 3*A*). Lastly, dysregulation of transcriptional networks in TFEB silenced MDA-MB-231 cells was interrogated using Enrichr to test for enrichment in gene sets derived from ChIP-X experiments listed in the ChEA database (34). This analysis identified that genes downregulated by TFEB knockdown were most associated with transcriptional regulation by FOXM1, MYC, and the E2F family (Fig. 3*B*). Of note, genes downregulated by TFEB knockdown are significantly associated with the gene set regulated by MITF in melanoma cells (Fig. 3*B*). These results suggest that TFEB cooperates with other transcription factors to regulate cell cycle gene expression in MDA-MB-231 cells.

**Table 1:** Motif enrichment identifies transcription networks downregulated by TFEB knockdown

Motif name	*p*-value	q-value (Benjamini)	# Of sequences with motif	% Of sequences with motif	% Of background sequences with motif	Enrichment ratio
NFY(CCAAT)	0.00	0.0287	198	28.78%	22.40%	1.284821429
BATF(bZIP)	0.00	0.0695	49	7.12%	4.28%	1.663551402
Hoxc9 (Homeobox)	0.01	0.1218	35	5.09%	2.97%	1.713804714
Atf3(bZIP)	0.01	0.1218	48	6.98%	4.48%	1.558035714
AP-1(bZIP)	0.01	0.1331	52	7.56%	5.01%	1.508982036
Fra1(bZIP)	0.01	0.1331	42	6.10%	3.88%	1.572164948
Fos(bZIP)	0.01	0.1331	43	6.25%	4.00%	1.5625
Bach1(bZIP)	0.01	0.1331	8	1.16%	0.36%	3.222222222
c-Myc(bHLH)	0.01	0.1331	65	9.45%	6.75%	1.4
Bach2(bZIP)	0.01	0.1331	20	2.91%	1.52%	1.914473684
E2F7(E2F)	0.10	0.1668	67	9.74%	7.35%	1.325170068
Tcfcp2l1(CP2)	0.10	0.1668	24	3.49%	2.10%	1.661904762
NFE2L2(bZIP)	0.10	0.197	6	0.87%	0.29%	3
Nrf2(bZIP)	0.10	0.2079	6	0.87%	0.30%	2.9
p53(p53)	0.10	0.2079	4	0.58%	0.15%	3.866666667

Genes downregulated by TFEB knockdown in MDA-MB-231 cells identified by RNA-Seq analysis were subjected to known motif enrichment using HOMER. Enriched promoters are displayed along with the statistical significance and the magnitude of enrichment.

### Knockdown of TFEB impairs S-phase entry

**Figure 4:**
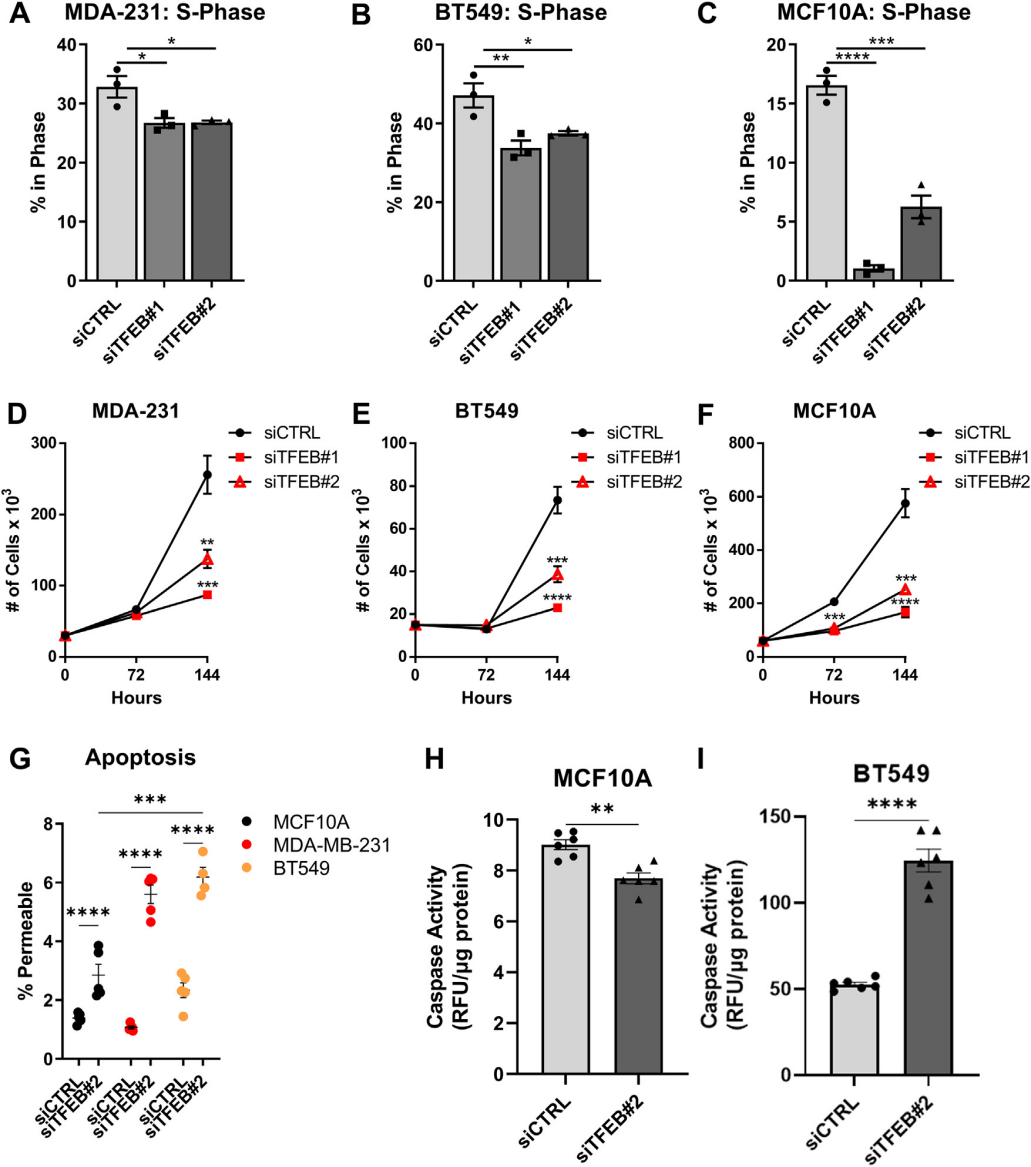
**TFEB knockdown reduces cell proliferation**. *A–C*, EdU cell cycle analysis results depict the percentage of cells in the S-phase following 72 h of TFEB knockdown in the indicated cell lines. *D–F*, cell counts at the indicated time points following TFEB knockdown in MDA-MB-231, BT549, and MCF10A cells. *G*, percent of cells that were permeable 120 h after TFEB knockdown in the indicated cell lines. *H*–*I*, caspase 3/7 activity 96 h after TFEB knockdown in the indicated cell line, depicted as fluorescence intensity corrected to the protein content. ∗*p* < 0.05, ∗∗*p* < 0.01, ∗∗∗*p* < 0.001, ∗∗∗∗*p* < 0.0001, one-way ANOVA (*A–G*), *t* test (*H–I*). TFEB, transcription factor EB.

To examine if regulation of cell cycle genes by TFEB contributes to altered cell function, cell cycle analysis was conducted by combining DNA content fluorescence quantification with the measurement of EdU incorporation to label S-phase cells. In both MDA-MB-231 and BT549 cells, knockdown of TFEB (Fig. S2, *A*–*F*) reduced the relative number of cells in the S-phase by ∼20% compared to the transfection control (Fig. 4, *A* and *B*). In noncancerous MCF10A cells, the effect of TFEB on cell cycle distribution was greater, with knockdown causing a ∼60% reduction in the percentage of S-phase cells compared to control (Fig. 4*C*). In agreement with cell cycle analysis, it was found that knockdown of TFEB reduced cell proliferation as measured by cell counting. In both MDA-MB-231 and BT549 cells, silencing of TFEB did not change cell numbers 72 h after treatment; however, after 144 h of knockdown, the cell count was significantly decreased by 2.5-fold compared to the nontargeting control siRNA (Fig. 4, *D* and *E*). Cell counting results in MCF10A cells similarly reflected the cell cycle analysis, with knockdown of TFEB significantly reducing cell numbers at both 72- and 144-h following treatment with siRNA (Fig. 4*F*). Cell death could also explain reduced cell numbers after TFEB knockdown; thus, the levels of cell death in TFEB silenced cells were quantified. In both MDA-MB-231 and BT549 cells, the level of cell death 120h hours following TFEB knockdown was increased five-fold and three-fold, respectively, compared to control, while in MCF10A cells, TFEB silencing increased cell death two-fold (Fig. 4*G*). Likewise, in BT549 cells, knockdown of TFEB significantly increased caspase activity by three-fold at 96 h; however, in MCF10A cells, knockdown of TFEB caused a slight decrease in caspase activity (Fig. 4, *H* and *I*). Together, these results validate that TFEB action regulates the cell cycle. Knockdown of TFEB in TNBC cell lines results in decreased numbers of S-phase cells, reduced cell proliferation, and increased caspase-dependent cell death. In contrast, TFEB knockdown does not result in cell death in noncancerous MCF10A cells but causes a significant decrease in the number of cells undergoing DNA replication.

**Figure 5:**
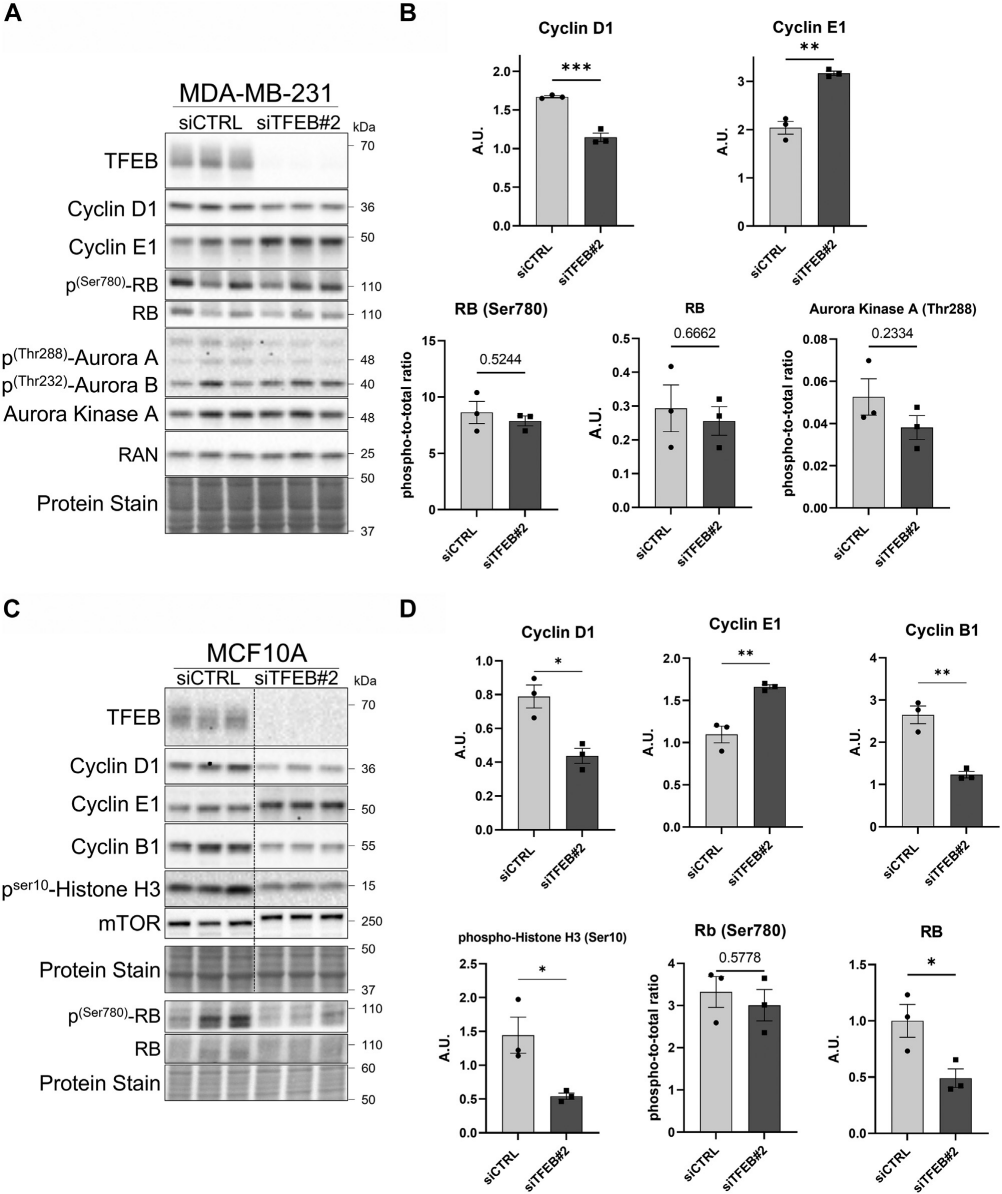
**TFEB silencing alters the level of G1/S regulatory proteins**. *A* and *B*, immunoblots and quantification of the indicated proteins in MDA-MB-231 cells following 72 h of TFEB knockdown. *C* and *D*, immunoblots and quantification of the indicated proteins in MCF10A cells following 72 h of TFEB knockdown. ∗*p* < 0.05, ∗∗*p* < 0.01, ∗∗∗*p* < 0.001, *t* test. TFEB, transcription factor EB.

Next, MDA-MB-231 cells treated with siRNA targeting TFEB or nontargeting control were subjected to immunoblot analysis to understand if TFEB knockdown altered the cell cycle at the protein level. Knockdown of TFEB reduced levels of the G1/S transition marker cyclin D1 at 72 h after treatment but caused a significant increase in the levels of cyclin E, an early S-phase marker (Fig. 5, *A* and *B*). The levels of phosphorylated and total RB did not change at 72 h following TFEB knockdown; however, at 96 h, the levels of total RB did significantly decrease (Figs. 5, *A*, *B* and S3). Additionally, levels of the mitosis marker threonine 288-phosphorylated AURKA were reduced 96 h following TFEB knockdown (Fig. S3). Changes in the protein levels of cyclin D1, E1, and phospho-AURKA in MDA-MB-231 cells were replicated using shRNA-mediated knockdown of TFEB, which confirms that this effect is the result of TFEB silencing (Fig. S4). These results align with the cell cycle analysis, which shows that TFEB causes an impaired G1/S transition in MDA-MB-231 cells. The level of cell cycle proteins was also analyzed in MCF10A cells in the context of TFEB knockdown, which showed that cyclin D1 levels were decreased, together with a concomitant increase of cyclin E1, while the levels of G2/M markers cyclin B1 and phosphorylated histone H3 (Serine 10) were reduced (Fig. 5, *C* and *D*). Levels of total RB were also decreased 72 h following TFEB knockdown in MCF10A cells, a response previously associated with CDK4/6 inhibition (35, 36). Therefore, in MDA-MB-231 and MCF10A cells, the protein expression pattern is consistent with impaired progression into the S-phase.

**Figure 6:**
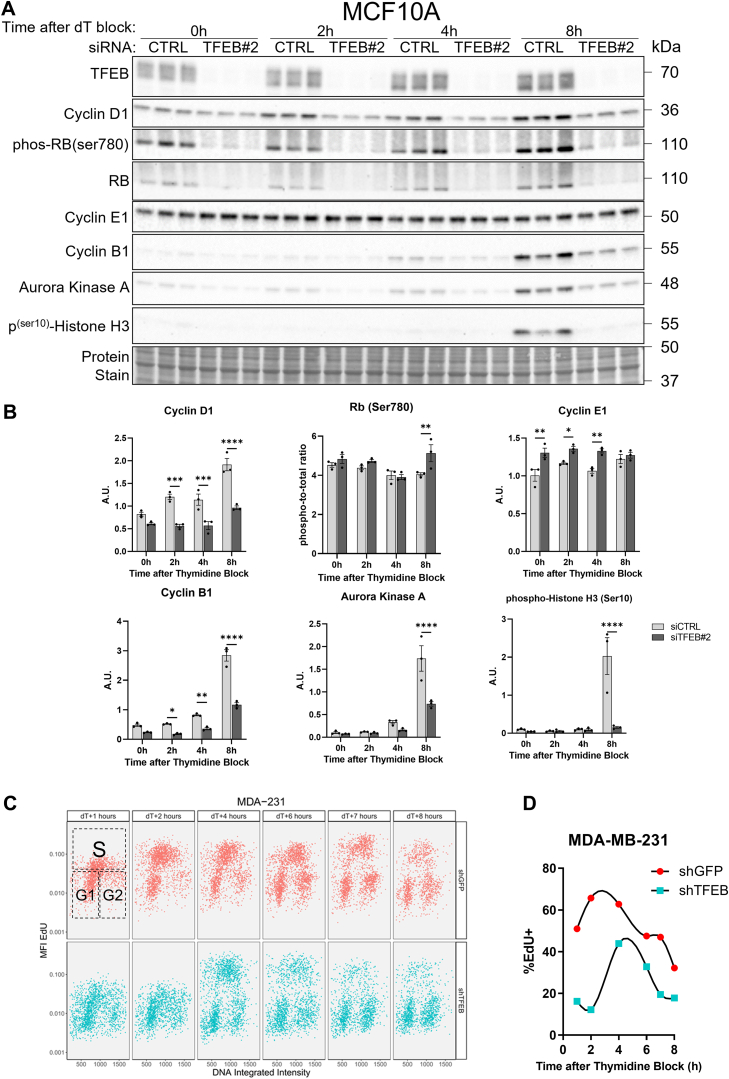
**TFEB knockdown results in G1/S arrest**. *A* and *B*, immunoblots and quantification from MCF10A cells with or without knockdown of TFEB at the indicated time points following synchronization at the G1/S transition through incubation for 18 h with 2 mM thymidine, an 8-h incubation in normal growth media, followed by a second incubation with 2 mM thymidine for 18 h ∗*p* < 0.05, ∗∗*p* < 0.01, ∗∗∗*p* < 0.001, ∗∗∗∗*p* < 0.0001, two-way ANOVA. *C*, EdU cell cycle analysis of TFEB knockdown MDA-MB-231 cells synchronized at the G1/S transition by 24 h of thymidine block and grown in the absence of thymidine for the indicated time points. *D*, the percentage of cells which entered the S-phase (EdU+) at the indicated times following thymidine block. TFEB, transcription factor EB.

To confirm whether TFEB knockdown was causing G1/S arrest and reduced progression through the S-phase, cells were treated with siRNA targeting TFEB, synchronized at the G1/S transition by double thymidine block, and released for time points between 0 and 8 h. In MCF10A cells, thymidine block significantly decreased protein markers of the G2 and M-phases at time points between 0 and 4 h after release; however, a sharp increase in cyclin B1, AURKA, and phospho-histone H3 at 8 h after the block signified progression through the S-phase into the M-phase (Fig. 6, *A* and *B*). In contrast, at 8 h following release from thymidine block, TFEB-knockdown MCF10A cells displayed a significant reduction in the protein levels of cyclin B1, AURKA, and phospho-Histone H3 compared to the control (Fig. 6, *A* and *B*). Therefore, in MCF10A cells, TFEB knockdown eliminates progression through the S-phase following thymidine block.

The effect of TFEB on S-phase progression was also quantified using thymidine block and EdU uptake in MDA-MB-231 cells. TFEB knockdown decreased the percentage of cells entering the S-phase at 1 h following thymidine block release from 50% to 16%, and at 2 h, from 65% to 12% (Fig. 6, *C* and *D*). In the control group 2 h after release from thymidine block, the percentage of cells in the S-phase peaks at 65%; however, the percentage peaks at 44% in cells with TFEB knockdown, 4 h following release (Fig. 6, *C* and *D*). These results confirm that loss of TFEB expression significantly hinders entry into the S-phase and the process of DNA replication in both MCF10A and MDA-MB-231 cells, which contributes to TFEB knockdown induced loss of cell proliferation.

### Loss of RB function exacerbates TFEB knockdown induced G1/S arrest and cell death

**Figure 7:**
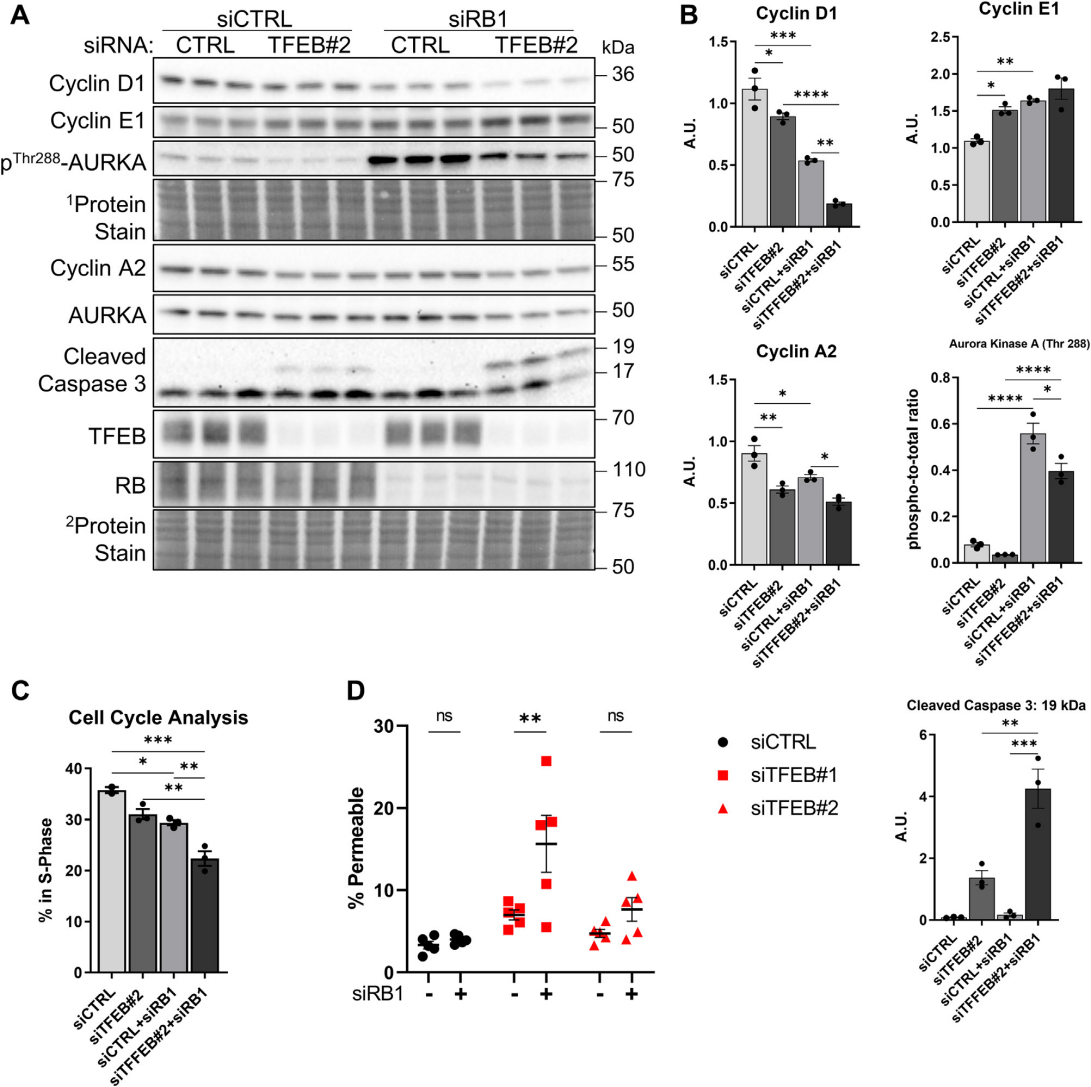
**Loss of RB1 function does not rescue G1/S arrest caused by TFEB knockdown.***A* and *B*, immunoblots and quantification of the indicated proteins from MDA-MB-231 cells treated with the indicated siRNAs for 72 h. *C*, percentage of cells in the S-phase quantified using EdU-DNA cell cycle analysis. *D*, percentage of dead cells determined by quantification of cell permeability. ∗*p* < 0.05, ∗∗*p* < 0.01, ∗∗∗*p* < 0.001, ∗∗∗∗*p* < 0.0001, one-way ANOVA, or two-way ANOVA (*D*). TFEB, transcription factor EB.

The results obtained suggest that TFEB knockdown results in reduced progression through the G1/S transition, therefore we questioned whether RB1, the suppressor which enforces the G1/S checkpoint, is required for this effect. In MDA-MB-231 cells, knockdown of RB1 significantly reduces protein levels of cyclin D1 and increases cyclin E1. Co-knockdown of TFEB and RB1 exacerbates the loss of cyclin D1 and increases cyclin E1 protein levels in an additive manner (Fig. 7, *A* and *B*). Similarly, cyclin A2 levels are decreased by both knockdown of RB1 and TFEB, while the combination of both siRNAs reduces cyclin A2 levels further (Fig. 7, *A* and *B*). In agreement with prior reports (37), we observed that phosphorylation of AURKA is elevated by knockdown of RB1; however, TFEB knockdown partially reverses this effect (Fig. 7, *A* and *B*). Furthermore, co-knockdown of TFEB with RB1 increased cleaved caspase-3 content, suggesting that the RB1-mediated G1/S checkpoint curbs induction of cell death caused by loss of TFEB expression (Fig. 7, *A* and *B*). In agreement with immunoblotting results, knockdown of TFEB significantly reduces the percentage of cells in the S-phase, and co-knockdown of TFEB and RB1 failed to rescue this decrease; rather, co-knockdown further reduced the number of cells in the S-phase in an additive manner (Fig. 7*C*). Lastly, knockdown of RB1 did not affect cell death in control cells; however, co-knockdown of RB1 with TFEB resulted in higher cell death rates than either treatment alone, although the increase was only significant with one siRNA targeting TFEB (Fig. 7*D*). Together, these results show that RB1 is not essential for the altered cell cycle protein expression and impaired S-phase entry that is caused by TFEB silencing. This finding suggests that the G1/S arrest induced by TFEB knockdown results from factors unrelated to G1/S checkpoint signaling.

### TFEB regulates replication origin licensing

**Figure 8:**
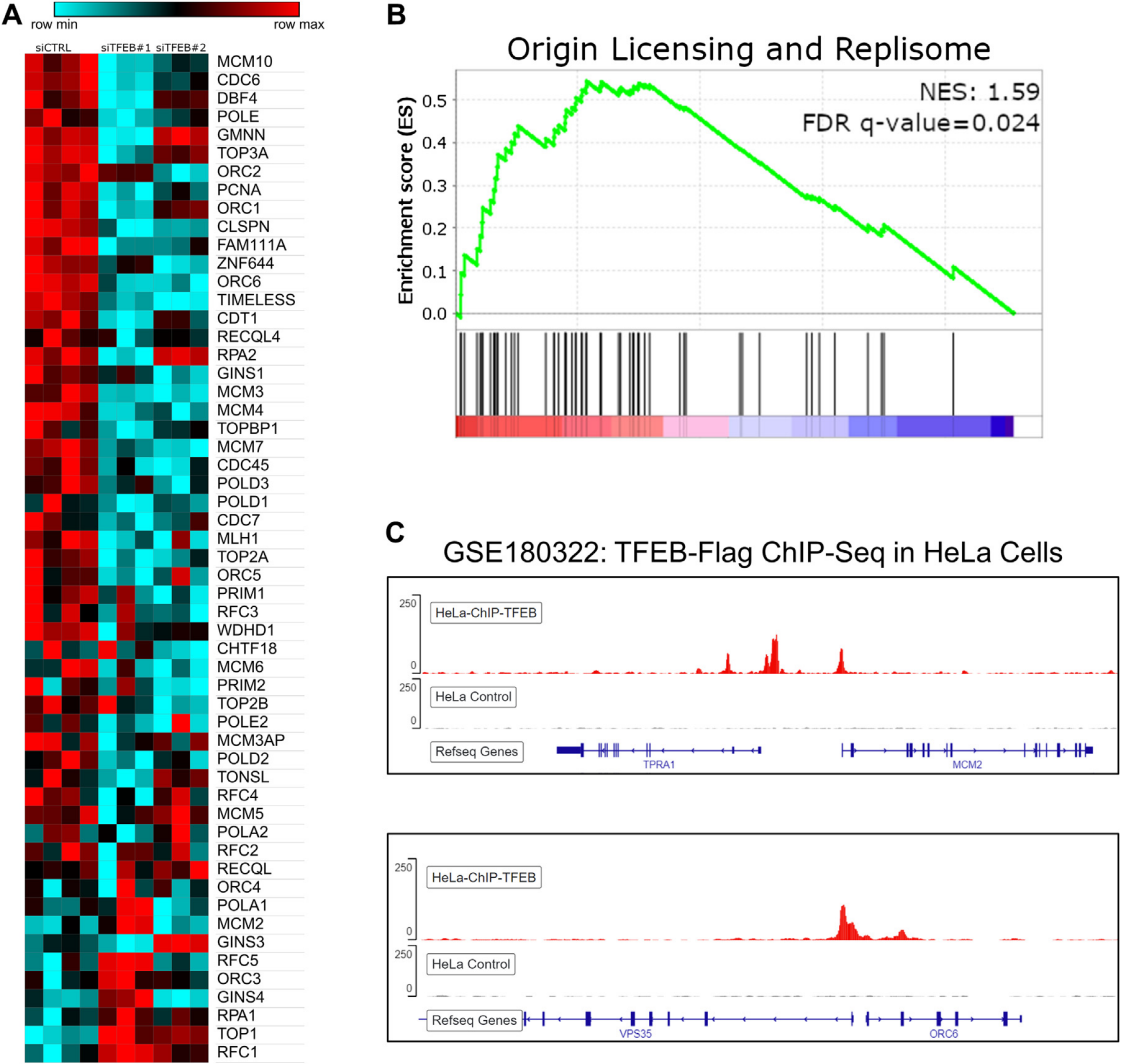
**Origin licensing and DNA replication genes are downregulated by TFEB knockdown.***A* and *B*, heatmap and enrichment plot for TFEB knockdown induced differential expression of genes involved in origin licensing and the replisome in MDA-MB-231 cells. *C*, ChIP-Seq peaks for Flag-TFEB (*red*) or wildtype control (*gray*) HeLa cells in the vicinity of the indicated genes, obtained from NCBI GEO series GSE1803222. TFEB, transcription factor EB.

Since knockdown of RB1 could not rescue the delayed S-phase entry in TFEB silenced MDA-MB-231 cells, we next investigated the function of replication origin licensing and DNA replication proteins. TFEB knockdown in MDA-MB-231 cells significantly decreased the expression of origin licensing genes, including CDC6, CDC45, ORC6, MCM3, MCM4, and MCM7 (Fig. 8*A*). Likewise, the gene set comprising the genes of origin licensing and replication showed significant downregulation following TFEB knockdown (Fig. 8*B*). Re-analysis of previously published, publicly available ChIP-Seq data from HeLa cells confirms a direct role for TFEB in regulating replication origin licensing gene expression (38). Flag-TFEB displayed elevated binding to the promoter regions of MCM2 and ORC6 in HeLa cells, indicating that TFEB likely transactivates the expression of these genes in human cells (Fig. 8*C*).

**Figure 9:**
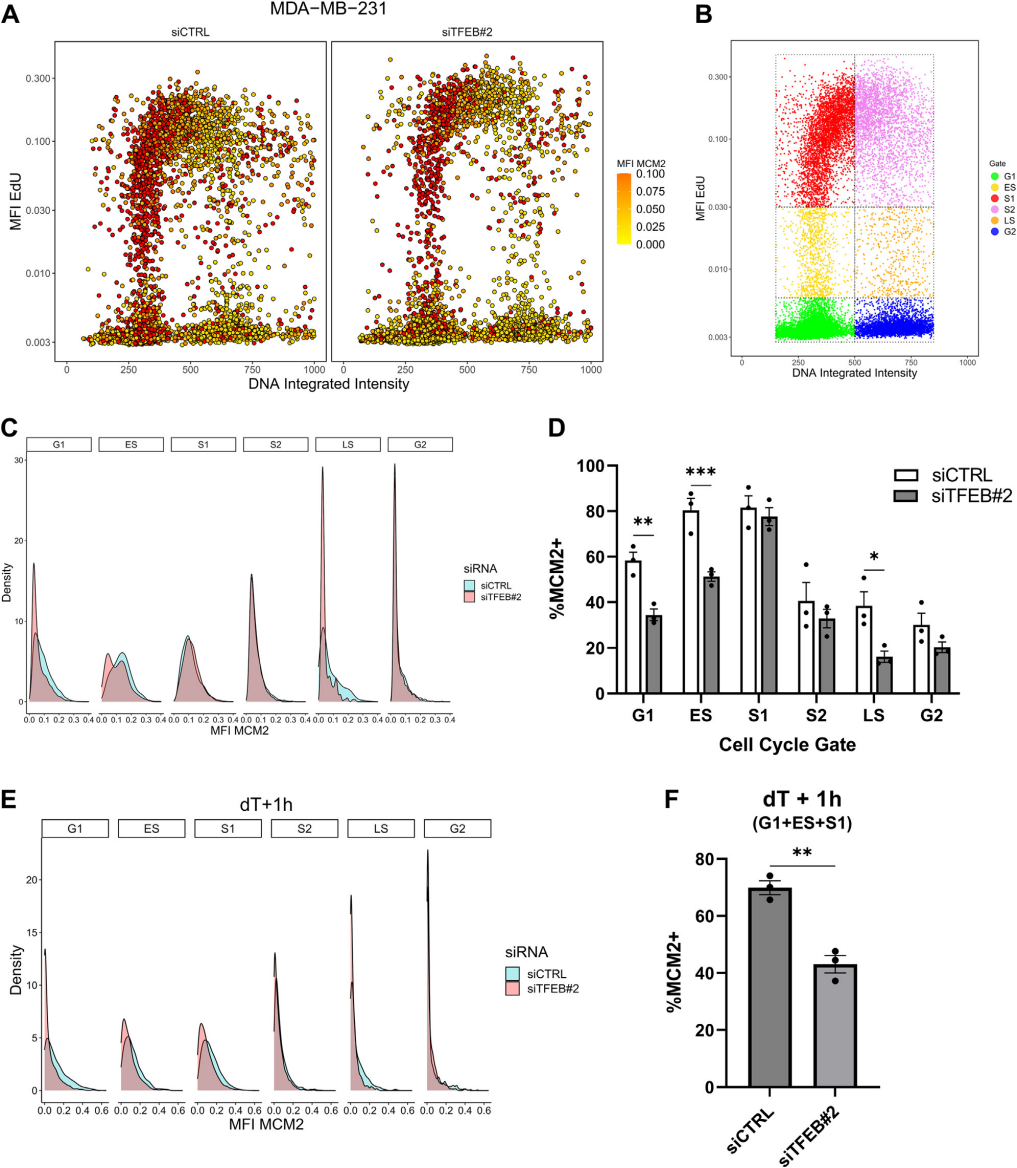
**TFEB silencing leads to origin under-licensing in MDA-MB-231 cells.***A*, imaging cytometry analysis of chromatin-bound MCM2 in MDA-MB-231 cells with or without TFEB knockdown, n = 6000 cells per treatment. *B*, gating strategy to assign cell cycle phases using EdU and DNA fluorescent intensities. *C*, smoothed density estimates for chromatin bound MCM2 levels by cell cycle gate as determined using EdU uptake and DNA content analysis. *D*, quantification of %MCM2 positive cells by cell cycle phase, n = 3 independent experiments. *E* and *F*, smoothed density estimate and MCM2 positivity by cell cycle phase from control or TFEB knockdown MDA-MB-231 cells released for 1 h following 24 h incubation with 2 mM thymidine, n = 3. ∗*p* < 0.05, ∗∗*p* < 0.01, ∗∗∗*p* < 0.001, (*C*) two-way ANOVA or (*E*) *t* test. TFEB, transcription factor EB.

To show that dysregulation of origin licensing gene expression by TFEB silencing corresponds with a functional defect in TNBC cells, we analyzed the cell cycle distribution of chromatin-bound MCM2 in TFEB knockdown MDA-MB-231 cells. In the control cells, chromatin-binding of MCM2 is highest in G1, early S, and mid-S-phase cells (termed hereafter as S1) before levels decrease with further DNA replication (Fig. 9, *A*–*D*). Knockdown of TFEB significantly reduces chromatin-bound MCM2 in G1 and early S-phase, with 20% fewer cells displaying MCM2 positivity in each of these phases (Fig. 9, *A*–*D*). Notably, the rate of MCM2 loading is equal between control and TFEB knockdown mid S-phase cells, and given that origin licensing is inhibited following the G1/S transition to prevent DNA re-replication, it is likely that this equalization is caused by the exit from DNA replication of under-licensed cells (Fig. 9, *A*–*D*). Origin licensing was also reduced by TFEB knockdown in BT549 cells as chromatin-bound MCM2 levels were significantly lower in the G1-Early S-S1 population of cells (Fig. S5, *A*–*C*).

A potential cause of origin under-licensing is a decreased time of G1, which forces cells into the S-phase before sufficient origins can be licensed (39). Moreover, cyclin E overexpression is associated with a shortened G1 time and origin under-licensing (39, 40). To determine if under-licensing in TFEB knockdown cells is caused by decreased G1 time, the time of G1 was extended through incubation with thymidine for 24 h, followed by analysis of MCM2 loading and cell cycle phase 1 h after release from the G1/S blockade. Following thymidine block, origin licensing remained suppressed in G1 and early S-phase TFEB knockdown cells, thus the time of G1 does not influence under-licensing after the loss of TFEB function (Fig. 9, *E* and *F*).

**Figure 10:**
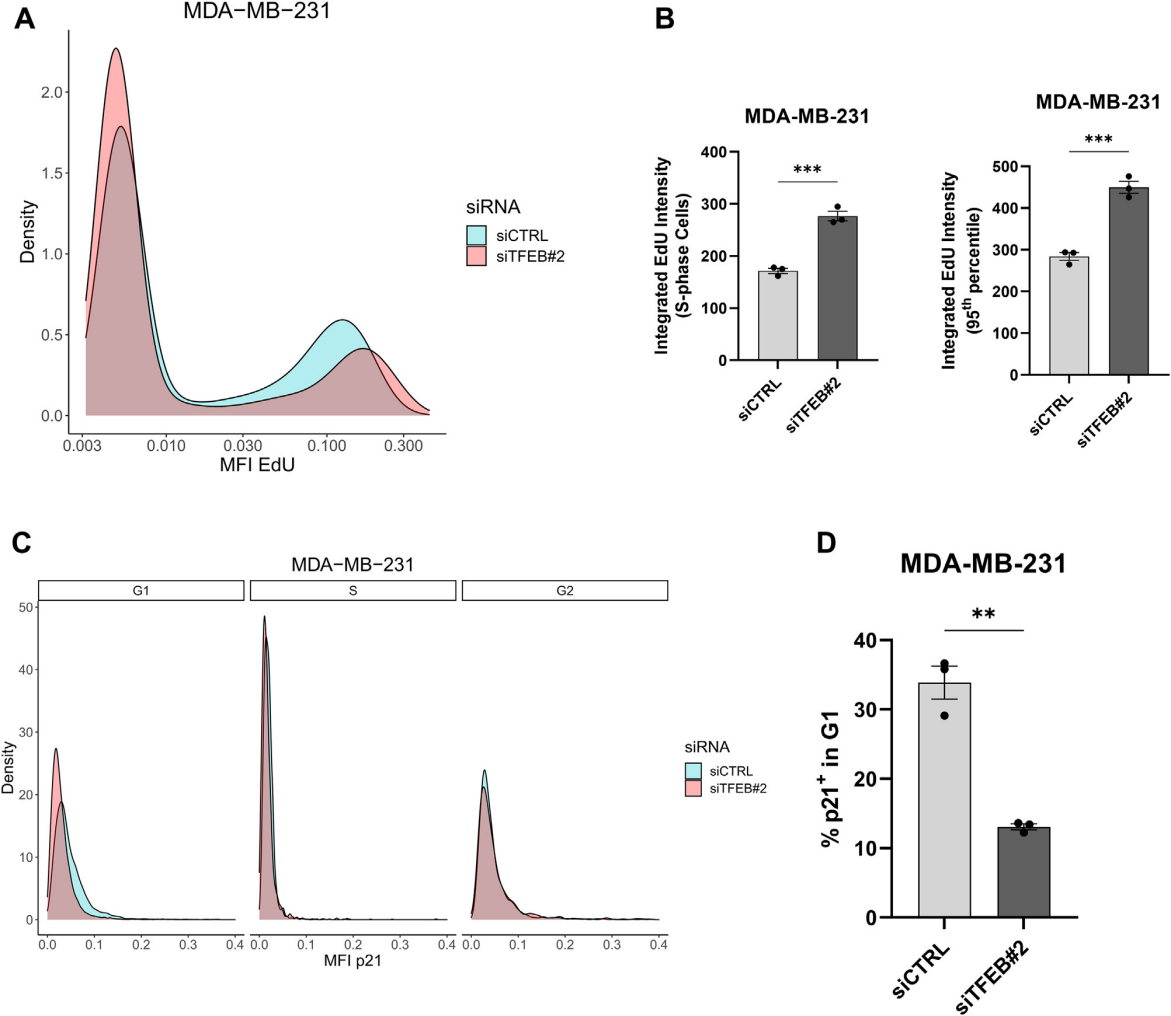
**TFEB knockdown elevates the rate of replication and decreases p21 expression.***A and B*, smoothed density estimate, quantification of total EdU fluorescence in S-phase cells, and the 95th percentile value for total EdU uptake, as a measure of maximal replication rate from control or TFEB knockdown MDA-MB-231 cells, n = 3. *C*, smoothed density estimates for p21 fluorescence intensity by cell cycle phase, as measured by imaging cytometry, for MDA-MB-231 cells treated with the indicated siRNA. *D*, quantification of the %p21 positive cells in G1 phase. ∗∗*p* < 0.01, ∗∗∗*p* < 0.001, *t* test. TFEB, transcription factor EB.

Prior reports show an association between reduced origin licensing and an overall increase in the speed of DNA replication (41). We find that TFEB knockdown likewise increases the rate of DNA replication in MDA-MB-231, as following 30 min of EdU uptake the total fluorescence intensity of EdU in the S-phase, along with the maximal (95th percentile) levels of EdU fluorescence were increased in TFEB knockdown cells compared to control (Fig. 10, *A* and *B*). Lastly, p53-mediated upregulation of p21 is hypothesized to prevent origin underlicensing, and TFEB has previously been identified as a regulator of p21 in cancer cells (42, 43). To understand if altered p21 expression could contribute to the licensing defect observed in TFEB depleted MDA-MB-231 cells, the levels of p21 were analyzed according to cell cycle phase using imaging cytometry. This analysis revealed that p21 expression was significantly suppressed in the G1 phase of TFEB knockdown cells, and thus, the ability to prevent S-phase entry of under-licensed cells is compromised (Fig. 10, *C* and *D*). In all, our results show that loss of TFEB function in MDA-MB-231 cells impairs proper origin licensing through the downregulation of critical origin licensing factors, and this effect impairs S-phase entry and cell proliferation.

### TFEB knockdown elevates markers of DNA damage and replication stress in MCF10A cells

Next, we questioned whether TFEB regulates origin licensing in noncancerous MCF10A cells. Analysis of MCM2 chromatin binding by cell cycle phase revealed a slight but insignificant decrease in early S-phase origin licensing resulting from TFEB knockdown, with no apparent change present in G1 (Fig. S6, *A*–*C*). Since TFEB knockdown did not alter origin licensing in MCF10A cells, we next examined other factors which might impair cell cycle progress under these conditions. A fundamental barrier to the efficient replication of DNA is DNA damage and replication stress (22). A marker of replication stress is endogenous DNA damage. Our prior results showed that knockdown of TFEB increases sensitivity to doxorubicin; however, we wanted to confirm whether knockdown alone induces DNA damage. In MCF10A cells, knockdown of TFEB with either of two siRNAs significantly elevated the formation of DNA damage, as indicated by γH2A.X foci, 96 h after treatment (Fig. S7, *A* and *B*). To quantify whether DNA damage resulted from replication stress in TFEB knockdown MCF10A cells, γH2A.X labeling was combined with EdU uptake and DNA staining to determine the level of DNA damage by cell cycle phase. In the control cells, DNA damage was significantly greater in both the S and G2 phases of the cell cycle (Fig. S7, *C* and *D*). However, following TFEB knockdown, γH2A.X levels in the S-phase were increased two-fold by both siRNA treatments compared to the control (Fig. S7, *C* and *D*). This result shows that DNA damage caused by TFEB knockdown occurs mainly in the S-phase of the cell cycle. Another measure of replication stress is the formation of chromatin-bound RPA (replication protein A) foci. RPA binds to single-stranded DNA during both replication stress and homologous recombination repair to increase the stability of the DNA strand (44). To detect chromatin-bound RPA70, soluble proteins are extracted from the cells prior to fixation, leaving only proteins bound to chromatin. Pre-extraction staining of RPA70 showed that knockdown of TFEB in MCF10A cells significantly increased the number of RPA70 foci per nuclei, indicating increased replication stress (Fig. S7, *E* and *F*). Together, these results show that TFEB knockdown elevates cell cycle associated DNA damage in proliferating noncancerous MCF10A cells.

Next, we tested whether TFEB silencing induces replication-associated DNA damage in TNBC cells. In BT549 and MDA-MB-231 cells, TFEB knockdown failed to significantly increase the levels of γH2A.X in the S-phase population beyond that found in control cells (Fig. S8, *A*–*D*). Likewise, TFEB knockdown alone was insufficient to elevate the level of chromatin-bound RPA70 in BT549 and MDA-MB-231 cells (Fig. S8, *E* and *F*). Lastly, we measured whether TFEB silenced TNBC cells were more sensitive to replication stress-inducing agents, such as hydroxyurea (HU). In MDA-MB-231 cells, 24 h of HU treatment significantly increased the number of chromatin-bound RPA70 foci; however, this increase was marginally blunted by silencing TFEB (Fig. S8*E*). In contrast, TFEB knockdown BT549 cells displayed significantly increased chromatin-bound RPA70 foci upon HU treatment compared to the transfection control (Fig. S8*F*). To summarize, TFEB silencing elevates markers of replication stress in noncancerous MCF10A cells but fails to significantly increase these markers in MDA-MB-231 or BT549 TNBC cells. However, knockdown of TFEB in BT549 cells significantly elevates RPA70 foci formation upon treatment with an inducer of replication stress.

### Kinase inhibitor screening identifies targetable vulnerabilities associated with loss of TFEB function

**Figure 11:**
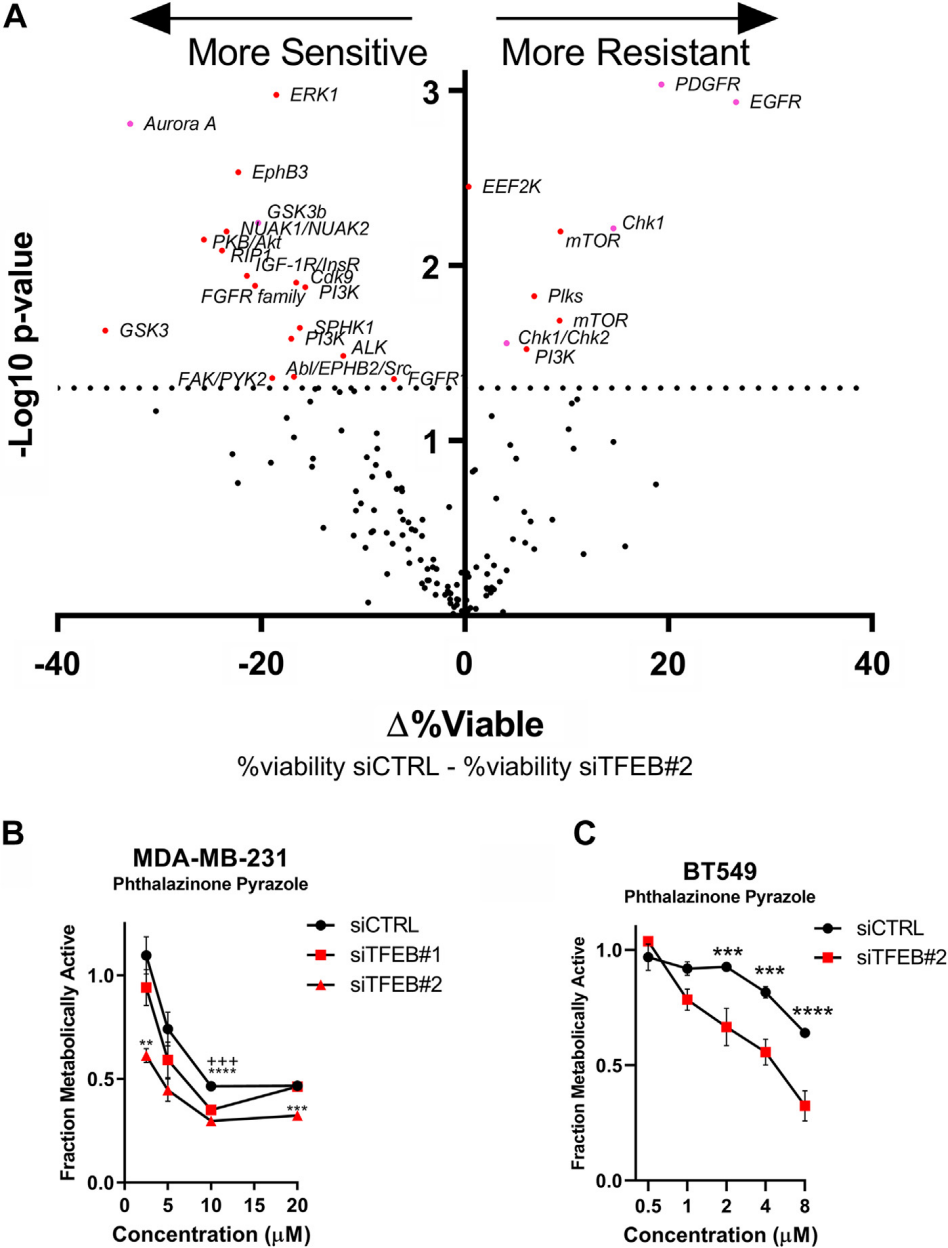
**Kinase inhibitor screening identifies synthetic lethality with TFEB knockdown and Aurora kinase A inhibition.***A*, volcano plot for the results of the kinase inhibitor screen, depicting the statistical significance and change in cell viability between siCTRL and siTFEB#2 transfected MDA-MB-231 cells following treatment with the indicated inhibitor for 72 h at 10 μM. *B* and *C*, metabolic fractional viability of TFEB knockdown MDA-MB-231 and BT549 cells treated with the indicated concentration of phthalazinone pyrazole for 72 h ∗∗*p* < 0.01, ∗∗∗*p* < 0.001, ∗∗∗∗*p* < 0.0001, one-way ANOVA. TFEB, transcription factor EB.

**Figure 12:**
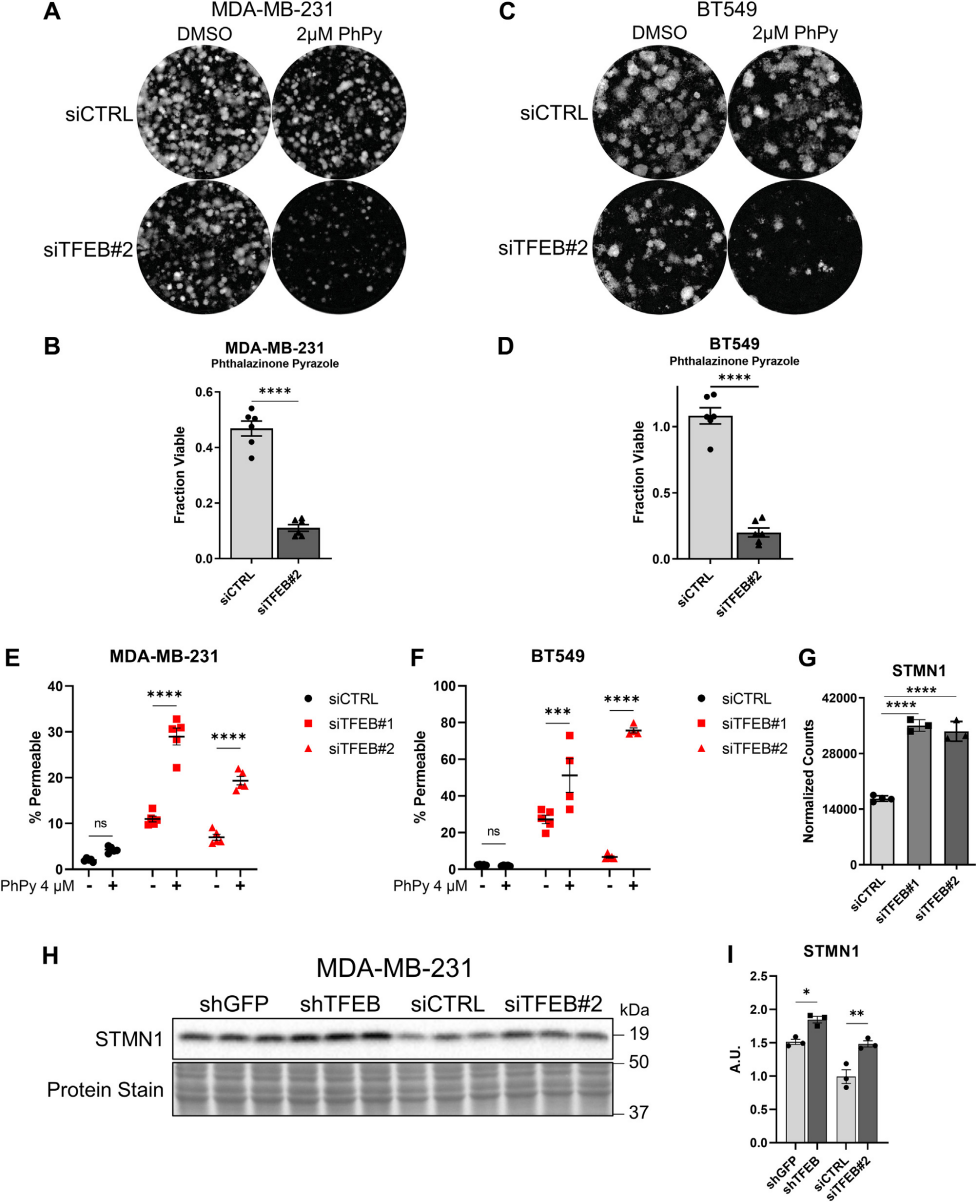
**Aurora kinase A inhibition significantly enhances TFEB knockdown induced cell death**. *A–D*, colony formation assay and quantification of siCTRL or siTFEB#2 transfected (*A* and *B*) MDA-MB-231 cells or (*C* and *D*) BT549 cells, n = 6 treatments from two independent experiments. *E* and *F*, percent cell death as quantified by cell permeability in the indicated cell lines following 72 h of treatment with 4 μM phthalazinone pyrazole, n = 4 or 5. *G*, RNA-Seq quantification of STMN1 gene expression from TFEB silenced MDA-MB-231 cells. *H* and *I*, immunoblot quantification of STMN1 protein expression in MDA-MB-231 cells 72 h after treatment with scramble control shRNA/siRNA or TFEB knockdown shRNA/siRNA. ∗*p* < 0.05, ∗∗*p* < 0.01, ∗∗∗*p* < 0.001, ∗∗∗∗*p* < 0.0001, *t* test (*B* and *D*), two-way ANOVA (*E* and *F*), one-way ANOVA (I). STMN1, Stathmin 1; TFEB, transcription factor EB.

We questioned whether cell cycle dysregulation caused by loss of TFEB function produced vulnerabilities that could be targeted by pharmacological inhibitors. Using the presto blue viability assay, 160 kinase inhibitors were screened, primarily targeting growth signaling, DNA damage response, and the cell cycle. In MDA-MB-231 cells, the inhibitor screen identified that TFEB knockdown rendered cells more resistant to compounds targeting CHK1/2, mTOR-PI3K signaling, and PDGFR/EGFR (Fig. 11*A*). Our data suggest that TFEB knockdown causes a relative reduction in response to these inhibitors by reducing cell viability through a common pathway. In contrast, TFEB knockdown increased the sensitivity to inhibitors of GSK3 and the AURKA inhibitor: phthalazinone pyrazole (PhPy) (Fig. 11*A*). Since the greatest change in viability between TFEB knockdown and control cells was seen with the AURKA inhibitor, and AURKA is important in mitotic progress, we chose to investigate this result further. In MDA-MB-231 and BT549 cells, TFEB knockdown significantly reduced cell viability in combination with doses of PhPy between 1 and 10 μM (Fig. 11, *B* and *C*). In addition, cell viability following PhPy treatment was assessed using colony formation assays. In both TNBC cell lines, knockdown of TFEB significantly sensitized cells to PhPy, with MDA-MB-231 and BT549 cells showing a 40% and 80% reduction in viability, respectively, relative to the control (Fig. 12, *A*–*D*). We tested whether the decrease in cell viability resulted from increased rates of cell death. Indeed, we found that the frequency of cell permeability was increased slightly by PhPy in MDA-MB-231 control cells, whereas PhPy increased cell permeability by over three-fold in both TFEB knockdown groups (Fig. 12*E*). A similar result was found in BT549 cells, where PhPy had no effect on control cells but increased the frequency of cell permeability to between 50 and 80% in cells treated with TFEB siRNA (Fig. 12*F*). Finally, a prior publication showed that upregulation of the microtubule depolymerizing protein Stathmin 1 (STMN1) in RB1 deficient lung cancer cells created synthetic lethality with AURKA inhibition (45). RNA-Seq transcriptomics found that TFEB silencing in MDA-MB-231 cells increased STMN1 gene expression by two-fold (Fig. 12*G*). Knockdown of TFEB using either shRNA or siRNA also elevated STMN1 protein levels in MDA-MB-231 cells; thus, STMN1 upregulation may explain synthetic lethality caused by TFEB knockdown and AURKA inhibition (Fig. 12, *H* and *I*). These findings indicate that AURKA is necessary for cell survival in the absence of TFEB function, and therefore combining inhibitors of TFEB with AURKA inhibitors may be a promising method to treat TNBC.

**Figure 13:**
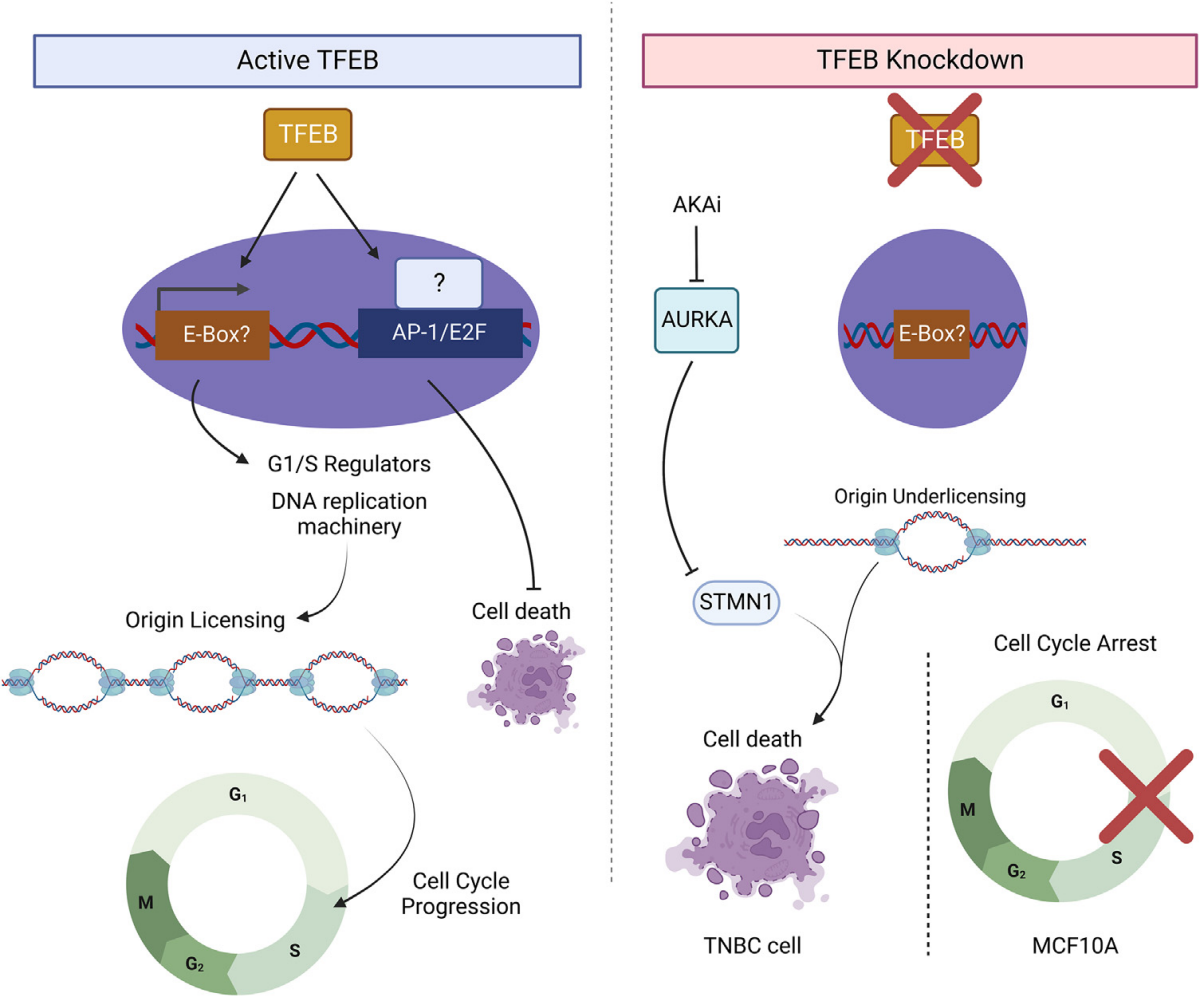
**Proposed model for TFEB mediated cell cycle regulation.** In proliferating cells, TFEB promotes the expression of G1/S regulators, DNA replication machinery, and origin licensing to ensure progression through the S-phase while inhibiting cell death. In contrast, knockdown of TFEB causes G1/S arrest or delay in S-phase entry and elevates the rate of cell death in association with origin under-licensing. In addition, STMN1 upregulation in TFEB knockdown cells may cause increased sensitivity to AURKA inhibition. AURKA, Aurora kinase A; STMN1, Stathmin 1; TFEB, transcription factor EB; TNBC, triple-negative breast cancer.

In summary, we find that TFEB supports cell proliferation in both TNBC and noncancerous breast epithelial cells. Silencing TFEB reduces the expression of DNA replication and mitosis genes and consequently suppresses the levels of cell cycle regulatory proteins in MDA-MB-231 and MCF10A cells. The number of cells undergoing DNA replication was decreased by TFEB knockdown, which was associated with G1/S arrest and the induction of apoptosis in cancer cell lines. Knockdown of RB1 could not rescue the decrease in cell proliferation caused by TFEB silencing. G1/S arrest induced by TFEB knockdown was accompanied by reduced origin licensing gene expression and decreased chromatin binding of MCM2 in G1 and the early S-phase. Lastly, we find that loss of TFEB function elevates sensitivity to AURKA inhibition (Fig. 13).

## Discussion

Dysregulation of the cell cycle is a hallmark feature of cancer, and TNBC is notable for showing elevated proliferation rates and expression of cell cycle genes (7, 18). Our prior results show that TFEB is likewise highly expressed in TNBC and silencing of TFEB in TNBC cell lines globally downregulates cell cycle gene expression. In this study, we see that TFEB contributes to the maintenance of TNBC cell proliferation and is necessary for the proliferation of non-cancerous MCF10A cells. These results led us to conclude that TFEB directly regulates the cell cycle; however, the mechanisms underlying this function remain unclear. Chromatin-immunoprecipitation sequencing experiments have found that TFEB directly promotes the expression of the G1/S regulator CDK4, transcriptional regulator CDK7, and replisome component MCM2 (14, 26, 27). Indeed, prior reports show that TFEB knockdown reduced CDK4 and RB1 phosphorylation levels in endothelial cells, which promoted G1/S arrest (26). These prior findings would explain G1/S arrest caused by TFEB knockdown; however, it was found that knockdown of the CDK4 target RB1 fails to rescue proliferation in MDA-MB-231 cells. Likewise, TFEB knockdown reduces EdU incorporation and cell proliferation in BT549 cells with a homozygous deletion of RB1. Therefore, the role of TFEB in the cell cycle is more significant than the regulation of the G1/S transition in TNBC cells. Many genes downregulated by TFEB knockdown are targets of canonical cell cycle regulating transcription factors such as MYC, E2F, and FOXM1 (46, 47, 48). Further study of how TFEB interacts with these transcription factors in TNBC is required to elucidate the mechanism behind cell cycle regulation by TFEB.

We propose that the TFEB-dependent regulation of origin licensing is a critical factor which leads to the impairment of cell proliferation in TNBC cells following TFEB knockdown. This effect could explain why cyclin E1 expression is higher following TFEB silencing, either as a compensatory mechanism to ensure firing of sufficient origins or as a consequence of under-licensed cells remaining arrested in the time following S-phase onset. The mechanism through which TFEB regulates origin licensing requires further study. Prior data indicate that MCM2 and ORC6 are transcriptional targets of TFEB, which we confirmed using publicly available ChIP-Seq data (14). Alternatively, the MiT/TFE family of transcription factors could participate in origin licensing directly, given that MITF is reported to interact with MCM3, MCM5, and MCM7 (49). Additionally, active promoter regions of chromatin are more easily licensed (50, 51), and thus loss of a transcription factor may render the chromatin less permissive to licensing. Finally, we cannot rule out that altered cell cycle regulatory networks contribute to under-licensing in TFEB knockdown cells by altering cell cycle phase lengths. Indeed, our data show that under-licensing in TFEB silenced cells is associated with reduced G1 phase p21 expression, which could remove the checkpoint that prevents S-phase entry before licensing can be complete. Under-licensing induced by TFEB knockdown may also explain the increased sensitivity of these cells to DNA damaging agents, as observed in our prior studies, given that diminished origin licensing capacity has recently been associated with elevated sensitivity to doxorubicin, camptothecin, and olaparib following long term treatment with CDK4/6 inhibitors (36).

In this study, we have not eliminated the possibility that TFEB knockdown-induced cell cycle impairment is a by-product of dysregulation in other critical pathways, such as metabolism, protein synthesis, or apoptosis. Indeed, in both MDA-MB-231 and BT549 TNBC cell lines, we find that the reduced cell number in TFEB knockdown groups manifests with decreased DNA replication and increased cell death. However, it is notable the RNA-seq analysis was conducted at 48 h following TFEB knockdown, before changes in cell cycle distribution and cell death begin to appear; therefore, the differential cell cycle gene expression is likely not a coincidental effect. Furthermore, silencing of TFEB in MCF10A cells causes G1/S arrest without activating apoptosis. These results suggest that direct regulation of the cell cycle is a key function of TFEB in proliferating cells. Since TFEB knockdown does not cause apoptosis in MCF10A cells, we propose a model wherein TFEB is necessary for cell survival in cancers containing certain oncogenic variations. Which TNBC genotypes render TFEB essential will be the subject of future research.

It is unclear whether replication stress plays a part in cell cycle arrest and cell death in TFEB knockdown cells. TFEB knockdown in MCF10A cells induces replication stress but not cell death; however, in TNBC cells, the reverse is true. Indeed, it is puzzling to find an increase in replication stress in MCF10A cells but not in TNBC cell lines, given that replication stress is often an outcome of replication origin under licensing. One possibility is that the TNBC cell lines studied could be more resistant to the development of replication stress. Further experimentation is required to untangle the relationship between TFEB and replication stress. Additional methods of DNA damage detection must be incorporated, such as the COMET assay.

An important difference between MCF10A and the TNBC cell lines is the presence of intact cell cycle checkpoints as regulated by p53 and RB1 (52). This genetic difference between cell lines may alter the fate of TFEB knockdown cells, where the lack of cell cycle control in MDA-MB-231 cells leads to origin under-licensing and impaired progression through the S-phase. In contrast, noncancerous MCF10A cells may be able to compensate for the loss of TFEB-directed gene expression and enforce cell cycle checkpoints to prevent S-phase entry. Indeed, prior studies have found that replication stress leads to G1 arrest in the daughter cells in a mechanism dependent on p53 and p21 (53, 54). Therefore, we propose that TFEB knockdown leads to G1 arrest in MCF10A cells through elevation of replication stress leading p53 activation. The causes of replication stress in TFEB knockdown cells will be the subject of future research.

Kinase inhibitor screening identified that inhibition of AURKA strongly increased cell death induced by TFEB silencing. AURKA has several roles in cellular function. During mitosis, AURKA localizes to centrosomes and spindle poles, where it is necessary for centrosome maturation and bipolar spindle assembly (55). Knockdown and inhibition of AURKA results in mitotic abnormalities, including multipolar spindle formation causing mitotic arrest and polyploidy (56). Additional roles of AURKA include stabilization of MYCN and regulating mitochondrial dynamics and function (57, 58). Prior studies have found that loss of the G1/S checkpoint through RB1 deletion causes synthetic lethality with AURKA inhibition. RB1 deletion hyperactivates the spindle assembly checkpoint, requiring high AURKA activity to prevent mitotic arrest and apoptosis (37). Subsequent studies have found that loss of RB1 renders lung cancer cells suspectable to microtubule destabilization due to overexpression of STMN1, a microtubule depolymerizing protein. AURKA inhibits STMN1, while inhibiting AURKA hyperactivates STMN1 in RB1-deficient cells, leading to mitotic cell death (45). Considering these findings, the role of TFEB in regulating mitotic processes and microtubule dynamics in TNBC will be the subject of further study. RNA-seq results do show that STMN1 is upregulated with TFEB knockdown by two-fold. Likewise, co-knockdown of RB1 with TFEB elevated cell death. In contrast, AURKA is involved in DNA fork protection during replication stress and regulation of homologous recombination, as such a role for AURKA in regulating genome stability in TFEB knockdown cells is possible (59).

In conclusion, we show that TFEB regulates the cell cycle in MDA-MB-231, BT549, and MCF10A cells, while loss of TFEB promotes cell cycle arrest, replication origin under-licensing, and sensitivity to AURKA inhibitors. These findings expand on the function of TFEB as an oncogene and provide a rationale for co-targeting TFEB and AURKA in TNBC patients.

## Experimental procedures

### Cell lines, transfections, transductions, and treatments

Culture, transfection, and transduction of MCF10A, MDA-MB-231, and BT549 cells were conducted as per methods described previously (10). The siRNAs used in this study were Ambion silencer select siRNA oligonucleotides (Thermo-Fisher Scientific): siTFEB#1: #s15495, siTFEB#2: #s15496, siRB1: #s522; siRNA negative control Cat# 4390844. For co-knockdown experiments, nontargeting control siRNA was added to the single knockdown groups (*i.e.*, siTFEB or siRB1 alone) to equalize siRNA concentration across treatments. In instances where only a single siRNA targeting TFEB was employed, siTFEB#2 targeting exon 7 was used since our prior data indicate it produces fewer off-target gene expression changes, as determined by RNA-Seq. Adenoviral delivery of shRNA targeting TFEB (Cat#: shADV-225358) or the control scrambled shRNA (Cat#: 1122) was accomplished using commercial constructs from Vector Biolabs.

Thymidine (dT) and HU were obtained from Millipore-Sigma and dissolved in water, and PhPy was obtained by Cayman Chemical and dissolved in DMSO. Double thymidine block was accomplished by incubating cells in 2 mM dT for 18 h, and then cells were washed once in growth media and cultured in thymidine free media for 8 h before another incubation for 18 h in 2 mM dT. A single thymidine block was used for imaging experiments, which consisted of incubation with 2 mM dT for 24 h. Cells were washed in growth media following thymidine block, then cultured in thymidine-free media for the indicated time points.

### RNA-seq and ChIP-Seq analysis

RNA-Seq transcriptomics analysis was conducted as described previously (10). Network analysis and visualization of cell cycle–related genes and gene sets were accomplished with Cytoscape (60). Promoter motif analysis was conducted using HOMER against the human genome (61), and identification of genes containing CLEAR sequences was accomplished by searching between −1000 and +100 base pairs relative to the transcription start site for the TCACGTGA motif. ChIP-Seq data for Flag-TFEB HeLa cells were obtained from NCBI GEO series GSE1803222 and visualized using IGV (Integrative Genomics Viewer, https://igv.org/app/) (62).

### Cell counting

Cell counting was conducted by manual counting with a hemocytometer. After 72 and 144 h of TFEB knockdown, cells were washed twice in PBS, with the wash solution collected each time. The attached cells were collected in media following a 5-min incubation in 0.05% trypsin 0.53 mM EDTA (Corning). The cells collected from trypsinization, and washing were combined and pelleted by centrifugation, then re-suspended in PBS. The cell concentration (viable plus nonviable) was determined by counting, and the total number of cells was obtained by multiplying the concentration with the volume.

### Cell viability and cell death assays

Colony formation assays, presto blue viability assays, and cell permeability assays were performed as described previously (10). Caspase activity was quantified with the Caspase-3 Activity Assay Kit (Cell Signaling Technologies) according to the manufacturer’s instructions. Cells were grown and treated in 96-well plates before being washed twice in PBS and lysed by incubation with Pathscan ELISA lysis buffer (Cell Signaling Technologies) for 5 min on ice. Lysates from two or three wells were combined with half used for caspase activity and half used for protein estimation. Cell lysate was combined with the substrate solution and incubated for 90 min in the dark before fluorescence intensity was read with a Synergy H4 plate reader, using 380 nM excitation and 440 nM emission. Protein concentration was obtained using the Pierce BCA Protein Assay Kit (Thermo Fisher Scientific) according to the manufacturer’s instructions. Data are represented as blank-corrected fluorescence intensity per μg of protein.

### Immunoblotting

Immunoblotting was conducted as per the methods described previously (10). Antibodies used in this study are listed in Table S1.

### Immunofluorescence

Immunofluorescence staining was conducted as described previously (10). For detection of chromatin-bound RPA70, media were aspirated, and cells were incubated with 0.2% Triton X-100–PBS on ice for 2 min, then fixed in 4% formaldehyde-PBS for 12 min before proceeding with the immunofluorescence protocol. For chromatin-bound MCM2 staining, cells on coverslips were pre-extracted in CSK buffer (100 mM NaCl, 300 mM sucrose, 3 mM MgCl_2_, 10 mM Pipes) + 0.5% Triton X-100 for 5 min on ice and then washed once in CSK buffer prior to fixation with in 4% formaldehyde-PBS for 12 min. Images presented in Fig. S7, and Fig. 8, *E* and *F* were acquired using a Zeiss LSM 900 with an Airyscan 2 detector at 20× magnification (20× Plan-Apochromat, NA: 0.8, air).

### High content imaging cell cycle analysis

EdU uptake was performed using the Click-iT EdU Cell Proliferation Kit for Imaging, Alexa Fluor 647 dye (Thermo Fisher Scientific), according to the manufacturer’s instruction. Briefly, cells on coverslips were incubated with 10 μM EdU for 30 min before fixation in 4% formaldehyde. Coverslips were washed in 3% BSA and permeabilized for 20 min using 0.2% Triton X-100–PBS. The click chemistry reaction time was 25 min. Subsequently, cells were washed with PBS, and then DNA stained by incubation with 1 μg/ml Hoechst 33342 for 2 min. Coverslips were mounted on slides using Prolong Gold Antifade Mountant (Thermo Fisher Scientific) and allowed to set for at least 48 h. When EdU uptake was combined with immunofluorescence, cells were permeabilized with 0.2% Triton X-100–PBS for 20 min and blocked for 45 min with 5% BSA before incubation with primary and secondary antibodies. Following antibody incubation, immunocomplexes were fixed with 4% formaldehyde-PBS for 5 min before proceeding with the click reaction. High content imaging was conducted by capturing 15 to 20 fields of view per coverslip with a Zeiss Axio Observer Z1 at 20× magnification (NA: 0.8, air). Images were processed with ImageJ using the subtract background function before nuclear intensities of EdU, Hoechst 33342, and γH2A.X were quantified with Cellprofiler (63, 64). Further normalization of intensity values, cell cycle phase determination, and data visualization was accomplished using custom R scripts. In brief, cells were labeled G1 or G2 if they were EdU-negative and had 2N or 4N DNA intensities, while cells were classified as in the S-phase if they were EdU-positive. Cellprofiler pipelines and R scripts used for processing of imaging data is available at: https://github.com/loganslade/JBC-Paper-2022.

### Kinase inhibitor screen

MDA-MB-231 cells were seeded in 96-well plates and treated with either nontargeting siRNA control or siRNA targeting TFEB for 48 h, then incubated with the Cayman Chemical kinase inhibitor library at a concentration of 10 μM per compound for 72 h in duplicate. After 72 h, media were aspirated and replaced with media containing presto blue, and then plates were incubated at 37 °C for 3 hours before fluorescence intensity was read with a Synergy H4 plate reader. Blank corrected fluorescence intensity was corrected to the siRNA specific DMSO control to quantify the relative viability change for each compound. Relative viability numbers were Log_2_ transformed and statistically analyzed with the R package limma (65).

### Gene expression analysis of breast cancer patient samples

Microarray log_2_ intensities and clinical data for breast cancer tumors from the METABRIC study were downloaded in October 2019 from cBioPortal (https://www.cbioportal.org/) (66). RNA-Seq normalized read counts, and clinical data for the The Cancer Genome Atlas breast cancer study were downloaded from the Firehose Broad GDAC portal (https://gdac.broadinstitute.org/) in January 2021, and PAM50 molecular subtypes for this study were obtained from the original manuscript (17). The data were processed and graphed using custom R scripts, which can be accessed at: https://github.com/loganslade/JBC-Paper-2022.

## Data availability

RNA-Seq data from TFEB knockdown MDA-MB-231 cells are deposited at NCBI GEO under the accession number GSE139203. TFEB-Flag ChIP-Seq data were obtained from NCBI GEO at the accession number GSE1803222. All other data available from the authors upon reasonable request.

## Supporting information

This article contains supporting information.

## Conflicts of interest

The authors declare that they have no conflicts of interest with the contents of this article.
