# Supplementary material for: Loss of transcription factor EB dysregulates the G1/S transition and DNA replication in mammary epithelial cells
Source: J Biol Chem. 2022 Nov 11;298(12):102692. doi: 10.1016/j.jbc.2022.102692 (PMC9764199; doi:10.1016/j.jbc.2022.102692)
Supplement: Supplemental data [file mmc1.pdf]

Supplementary Table 1. Supplier information for the antibodies used in this manuscript.

Reagent	Source	Identifier/Catalog#
Alexa Fluor 488 goat-anti-mouse	ThermoFisher	A11001
Alexa Fluor 488 goat-anti-rabbit	ThermoFisher	A11008
Alexa Fluor 594 goat anti-mouse	ThermoFisher	A11005
Alexa Fluor 594 goat anti-rabbit	ThermoFisher	A11012
Anti-mouse IgG, HRP-linked Antibody	Cell Signaling	7076S
Anti-rabbit IgG, HRP-linked Antibody	Cell Signaling	7074S
Aurora Kinase A	Cell Signaling	14475
Cleaved Caspase-3	Cell Signaling	9664
Cyclin A2	Cell Signaling	4656
Cyclin B1	Cell Signaling	12231
Cyclin D1	Cell Signaling	2978
Cyclin E1	Cell Signaling	4129
MCM2	Cell Signaling	3619
mTOR	Cell Signaling	2972
p21/CDKN1A	Cell Signaling	2947
PCNA	Santa Cruz	sc-56
Phospho-Aurora A (Thr288)/ Aurora B (Thr232)/ Aurora C (Thr198)	Cell Signaling	2914
phospho-H2A.X (Ser139)	Cell Signaling	2577
phospho-H2A.X (Ser139)	Cell Signaling	9718
phospho-H2A.X (Ser139)	Millipore	05-636-I
phospho-Histone H3 (Ser10)	Cell Signaling	3377
phospho-RB1 (Ser780)	Cell Signaling	8180
RAN-GTPase	BD Transduction Laboratories	610341

Supplementary table 2. Motif discovery identifies TFEB regulated genes in MDA-MB-231 cells. Genes downregulated by TFEB knockdown in MDA-MB-231 cells identified by RNA-Seq analysis were subjected to CLEAR promoter discovery using HOMER. Identified genes are displayed along with the promoter location relative to the transcription start site (offset), the promoter sequence, strand specificity, and similarity of the discovered promoter to the consensus sequence (MotifScore).

Offset	Sequence	Strand	MotifScore	Name
-189	CCACGTGA	-	8.5476	GK
-80	GCACGTGA	+	8.5476	FOXRED2
-289	TCACGTGC	-	8.5476	FOXRED2
-926	TCACGTGA	+	10.765	ZNF74
-919	TCACGTGA	-	10.765	ZNF74
-231	TCACGTGA	+	10.765	RCAN1
-224	TCACGTGA	-	10.765	RCAN1
-29	TCACGTGG	+	8.5476	ARFGEF2
-21	TCACGTGA	+	10.765	ARFGEF2
-14	TCACGTGA	-	10.765	ARFGEF2
-60	CCACGTGA	-	8.5476	STK4
-179	TCACGTGG	+	8.5476	DHX35
-127	TCACGTGG	+	8.5476	DHX35
-15	CCACGTGA	-	8.5476	ESF1
-564	TCACGTGA	+	10.765	WDR62
-467	TCACGTGA	+	10.765	WDR62
-460	TCACGTGA	-	10.765	WDR62
-557	TCACGTGA	-	10.765	WDR62
77	TCACGTGC	-	8.5476	CBARP
-615	CCACGTGA	-	8.5476	TAF4B
-16	TCACGTGG	+	8.5476	RMC1
-538	TCACGTGA	+	10.765	SECTM1
-531	TCACGTGA	-	10.765	SECTM1
-932	TCACGTGC	-	8.5476	ZNF207
-51	TCACGTGG	+	8.5476	ESRP2
-149	GCACGTGA	+	8.5476	PAQR5
-113	GCACGTGA	+	8.5476	OTUB2
-217	CCACGTGA	-	8.5476	GTF2A1
-441	CCACGTGA	-	8.5476	GTF2A1
-468	TCACGTGA	+	10.765	ALDH6A1
-461	TCACGTGA	-	10.765	ALDH6A1
-843	CCACGTGA	-	8.5476	TIMM9
-449	CCACGTGA	-	8.5476	STYX
-61	GCACGTGA	+	8.5476	TPP2
-829	TCACGTGC	-	8.5476	LNX2

Offset	Sequence	Strand	MotifScore	Name
28	GCACGTGA	+	8.5476	UTP20
-26	CCACGTGA	-	8.5476	UTP20
15	GCACGTGA	+	8.5476	NEMP1
-2	TCACGTGA	+	10.765	PRMT3
5	TCACGTGA	-	10.765	PRMT3
-700	CCACGTGA	-	8.5476	CNNM2
-58	TCACGTGA	+	10.765	SIRT1
-51	TCACGTGA	-	10.765	SIRT1
-130	CCACGTGA	-	8.5476	SIRT1
-72	TCACGTGA	+	10.765	NUP188
-25	TCACGTGG	+	8.5476	NUP188
-65	TCACGTGA	-	10.765	NUP188
-851	TCACGTGG	+	8.5476	GKAP1
-7	TCACGTGA	+	10.765	SLC25A32
0	TCACGTGA	-	10.765	SLC25A32
-34	CCACGTGA	-	8.5476	MTDH
-66	CCACGTGA	-	8.5476	MTDH
-551	TCACGTGA	+	10.765	DNAJB9
79	TCACGTGG	+	8.5476	DNAJB9
-544	TCACGTGA	-	10.765	DNAJB9
-21	CCACGTGA	-	8.5476	TRIP6
-686	TCACGTGG	+	8.5476	ARL4A
39	TCACGTGC	-	8.5476	SRFBP1
-687	TCACGTGG	+	8.5476	NOCT
-422	CCACGTGA	-	8.5476	BMP2K
-31	GCACGTGA	+	8.5476	THAP6
38	CCACGTGA	-	8.5476	RRP9
-304	CCACGTGA	-	8.5476	TSEN2
-140	TCACGTGC	-	8.5476	CCNYL1
-707	TCACGTGC	-	8.5476	AGPS
28	TCACGTGA	+	10.765	HNRNPA3
35	TCACGTGA	-	10.765	HNRNPA3
61	CCACGTGA	-	8.5476	METAP1D
89	TCACGTGG	+	8.5476	CAD
-119	CCACGTGA	-	8.5476	BATF3
-54	TCACGTGG	+	8.5476	PLEKHA6
-171	CCACGTGA	-	8.5476	CGN
-93	TCACGTGA	+	10.765	PIP5K1A
-86	TCACGTGA	-	10.765	PIP5K1A
-128	TCACGTGA	+	10.765	RBM15
-121	TCACGTGA	-	10.765	RBM15
9	TCACGTGA	+	10.765	COA7
16	TCACGTGA	-	10.765	COA7
-269	GCACGTGA	+	8.5476	DPH2

Offset	Sequence	Strand	MotifScore	Name
-130	TCACGTGC	-	8.5476	TENT5B

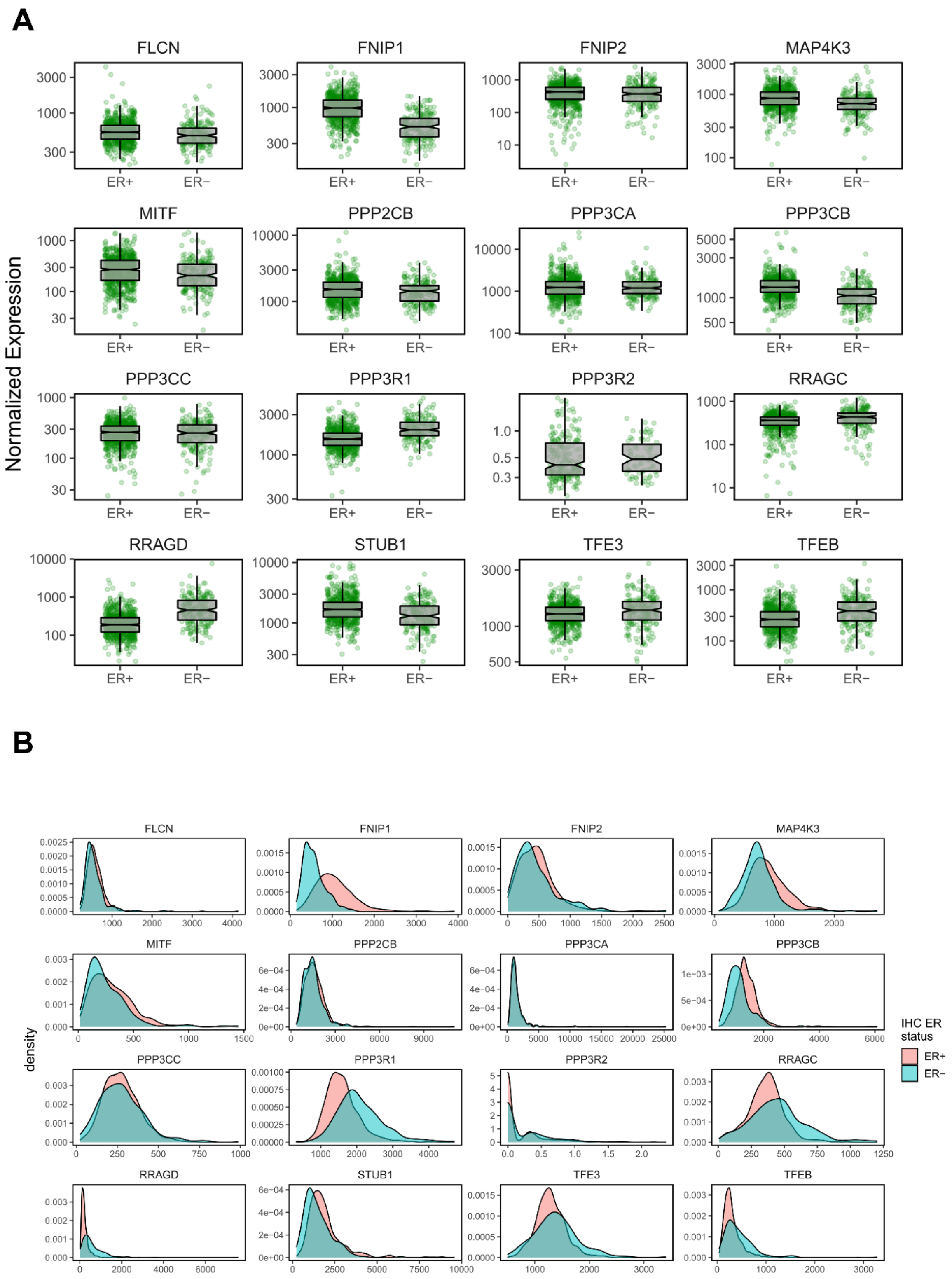

Supplementary Figure 1. Gene expression of TFEB regulators in ER+ and ER- breast cancer patients. (A) Boxplots or (B) smoothed density estimates of RSEM normalized gene expression values for genes which regulate the localization and function of TFEB, as measured by RNA-Seq from breast tumor biopsies collected by the TCGA: breast cancer study, separated by IHC estrogen receptor status.

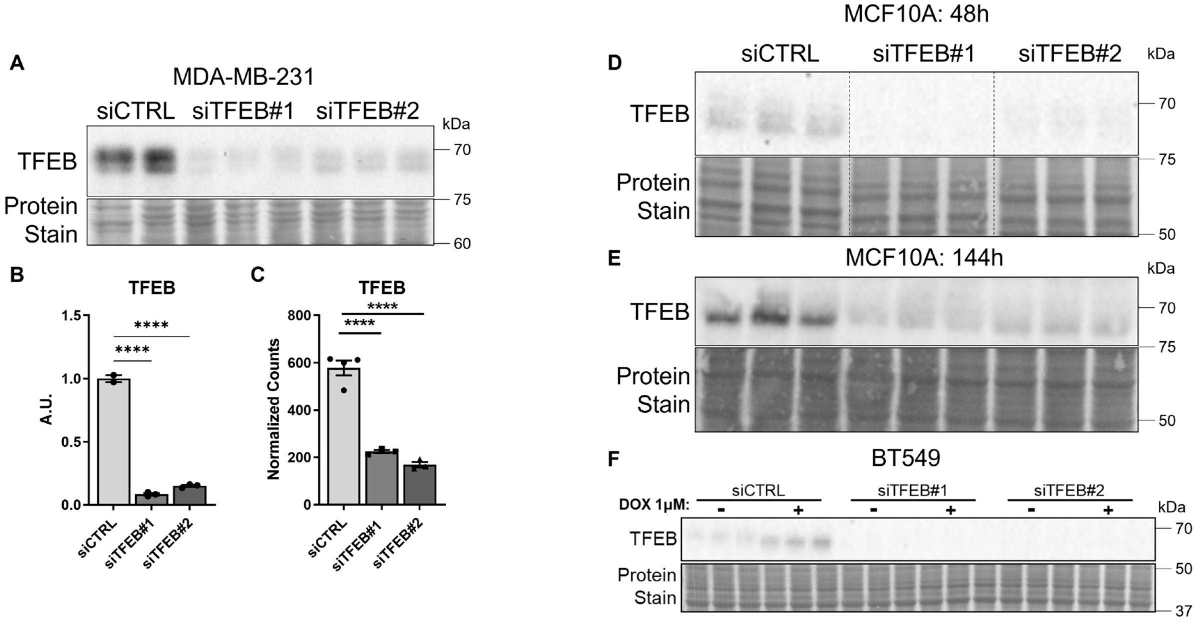

Supplementary Figure 2. Validation of siRNA mediated TFEB silencing. (A, B) Immunoblot and quantification of TFEB protein levels 48 hours following siRNA transfection in MDA-MB-231 cells. (C) DESeq2 normalized counts derived from MDA-MB-231 cells 48 hours following siRNA treatment, as quantified by RNA-Seq. (D, E) TFEB immunoblot from MCF10A cells 48- and 144-hours following siRNA transfection. (F) TFEB immunoblot from vehicle or doxorubicin treated BT549 cells 72 hours following siRNA transfection. Doxorubicin treatment used to elevate TFEB protein levels for the demonstration of knockdown efficacy. ****p<0.0001, one-way ANOVA.

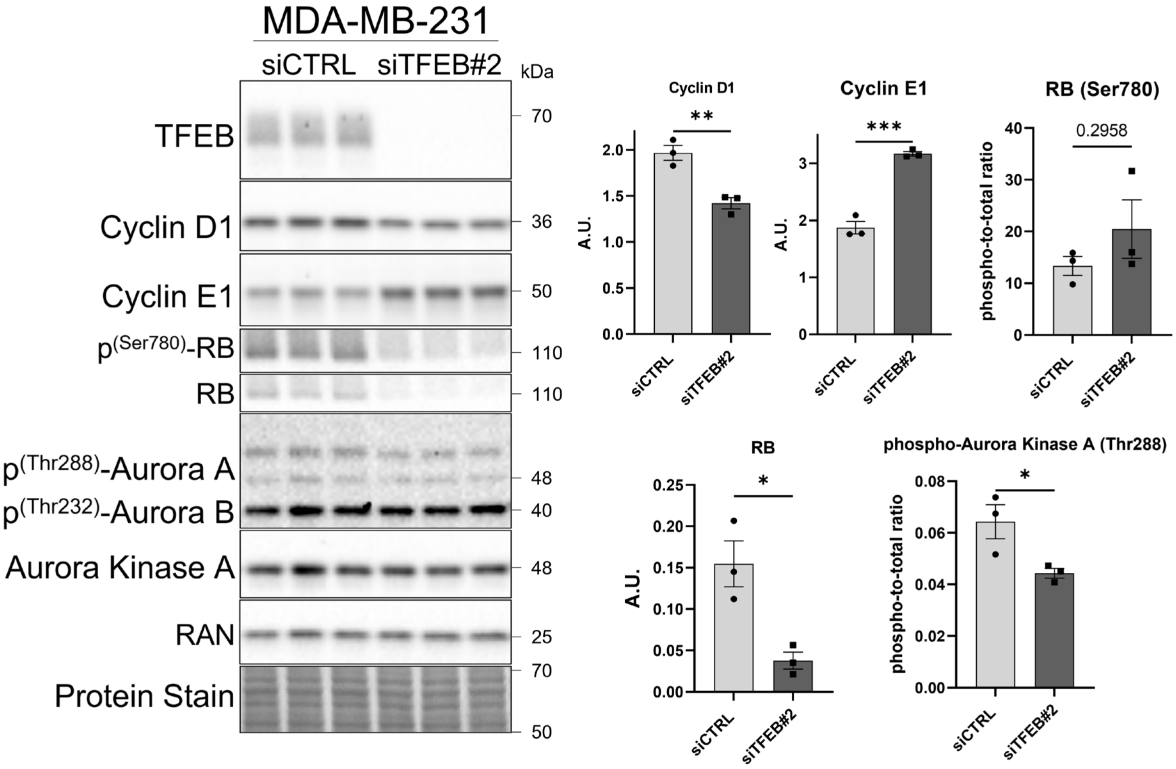

Supplementary Figure 3. G1/S protein levels are dysregulated 96 hours following TFEB knockdown in MDA-MB-231 cells. Immunoblots and quantification of the indicated proteins in MDA-MB-231 cells 96 hours after TFEB knockdown. *$p<0.05$, **$p<0.01$, ***$p<0.001$, t-test.

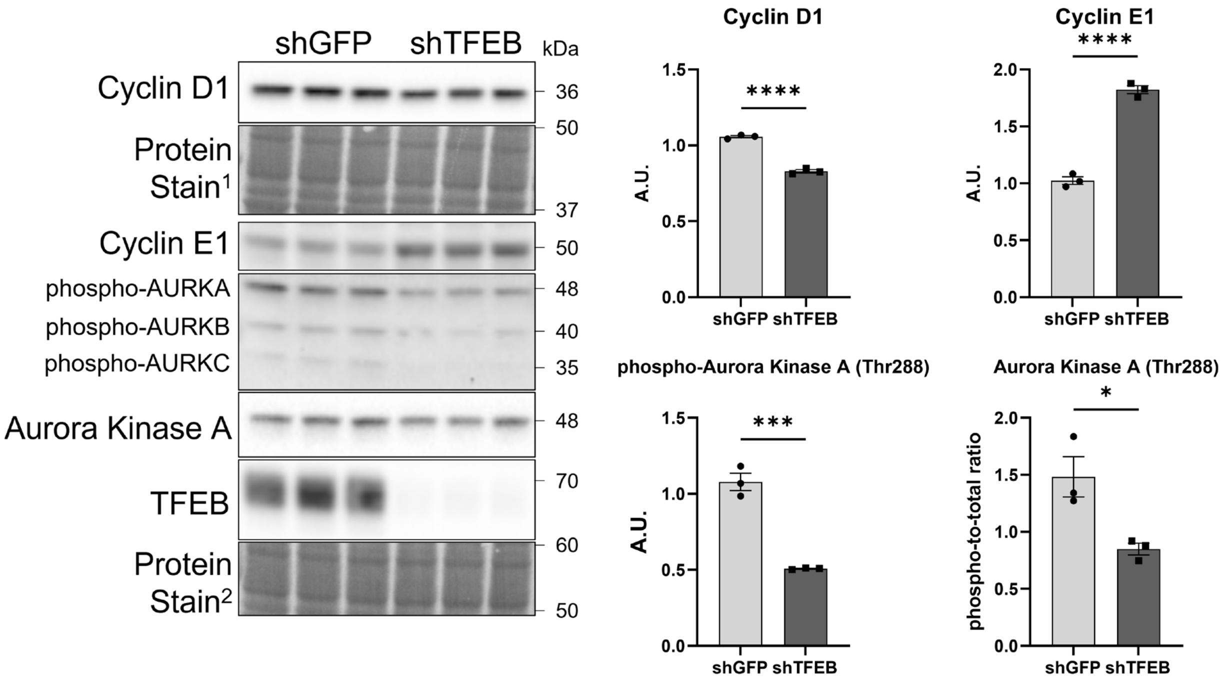

Supplementary Figure 4. G1/S protein levels are dysregulated by shRNA mediated TFEB knockdown in MDA-MB-231 cells. Immunoblots and quantification of the indicated proteins in MDA-MB-231 cells 72 hours after adenoviral shRNA transduction. *p<0.05, ***p<0.01, ****p<0.0001, t-test.

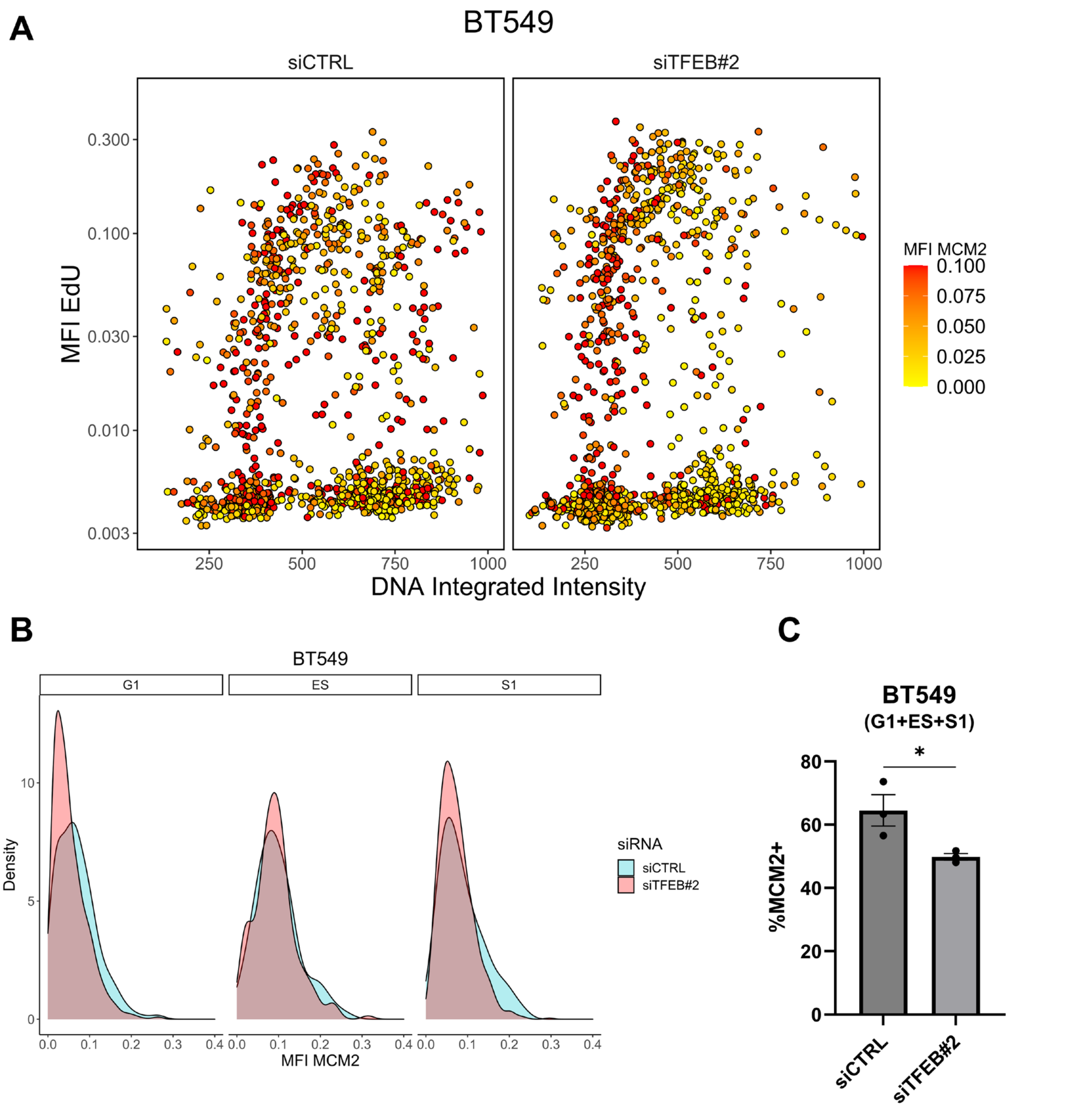

Supplementary Figure 5. TFEB silencing leads to origin underlicensing in BT549 cells. (A) Imaging cytometry analysis of chromatin bound MCM2 in BT549 cells with or without TFEB knockdown, n=1200 cells per treatment. (B) Smoothed density estimates for chromatin bound MCM2 levels by cell cycle gate as determined using EdU uptake and DNA content analysis. (C) Quantification of %MCM2 positive cells with 2N DNA (comprising the G1, early-S, and S1 gates), n=3 independent experiments. *$p<0.05$, t-test.

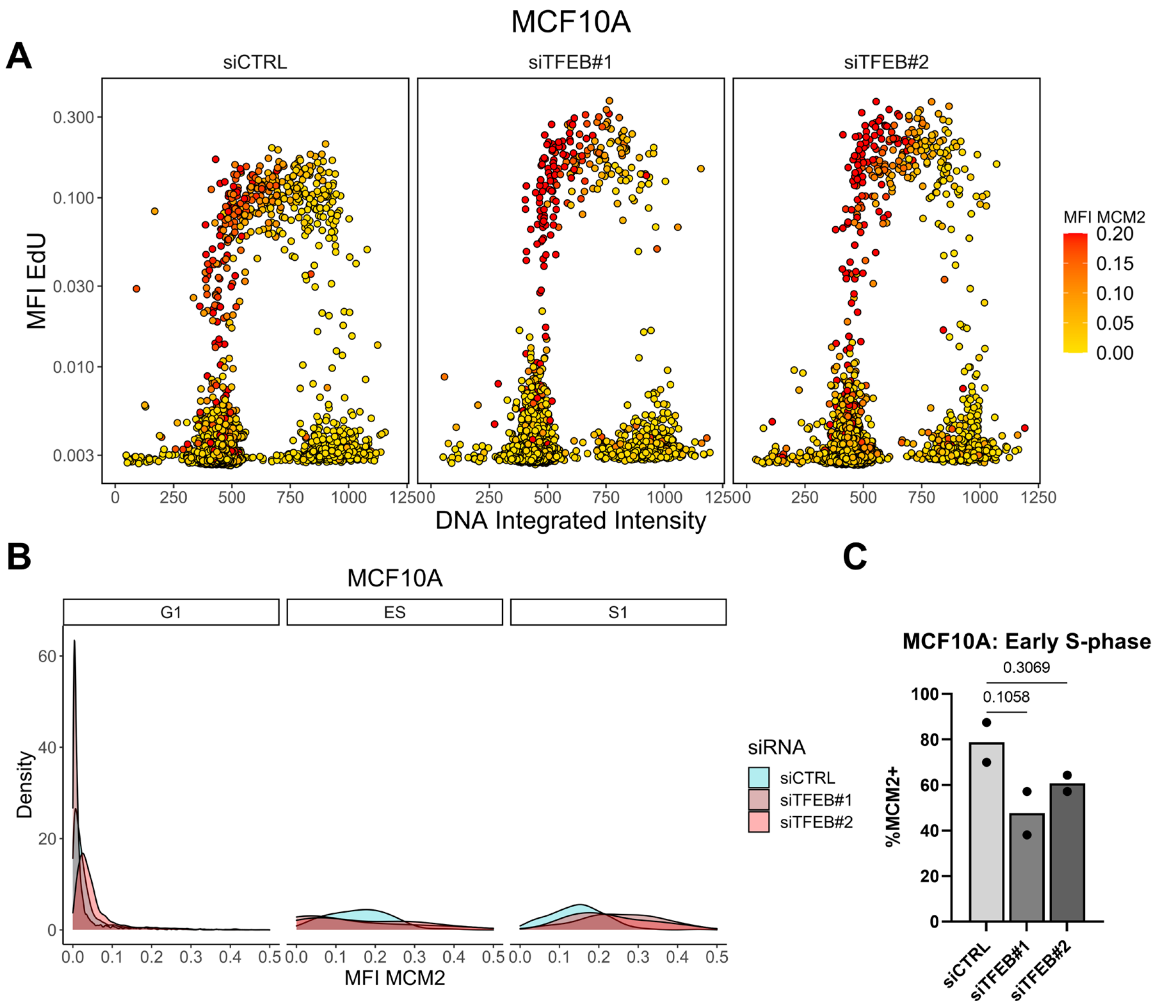

Supplementary Figure 6. TFEB knockdown does not alter origin licensing in MCF10A cells. (A) Imaging cytometry analysis of chromatin bound MCM2 in MCF10A cells with or without TFEB knockdown, n=4500 cells per treatment. (B) Smoothed density estimates for chromatin bound MCM2 levels by cell cycle gate as determined using EdU uptake and DNA content analysis. (C) Quantification of %MCM2 positive cells by cell cycle phase, n = 3 independent experiments.

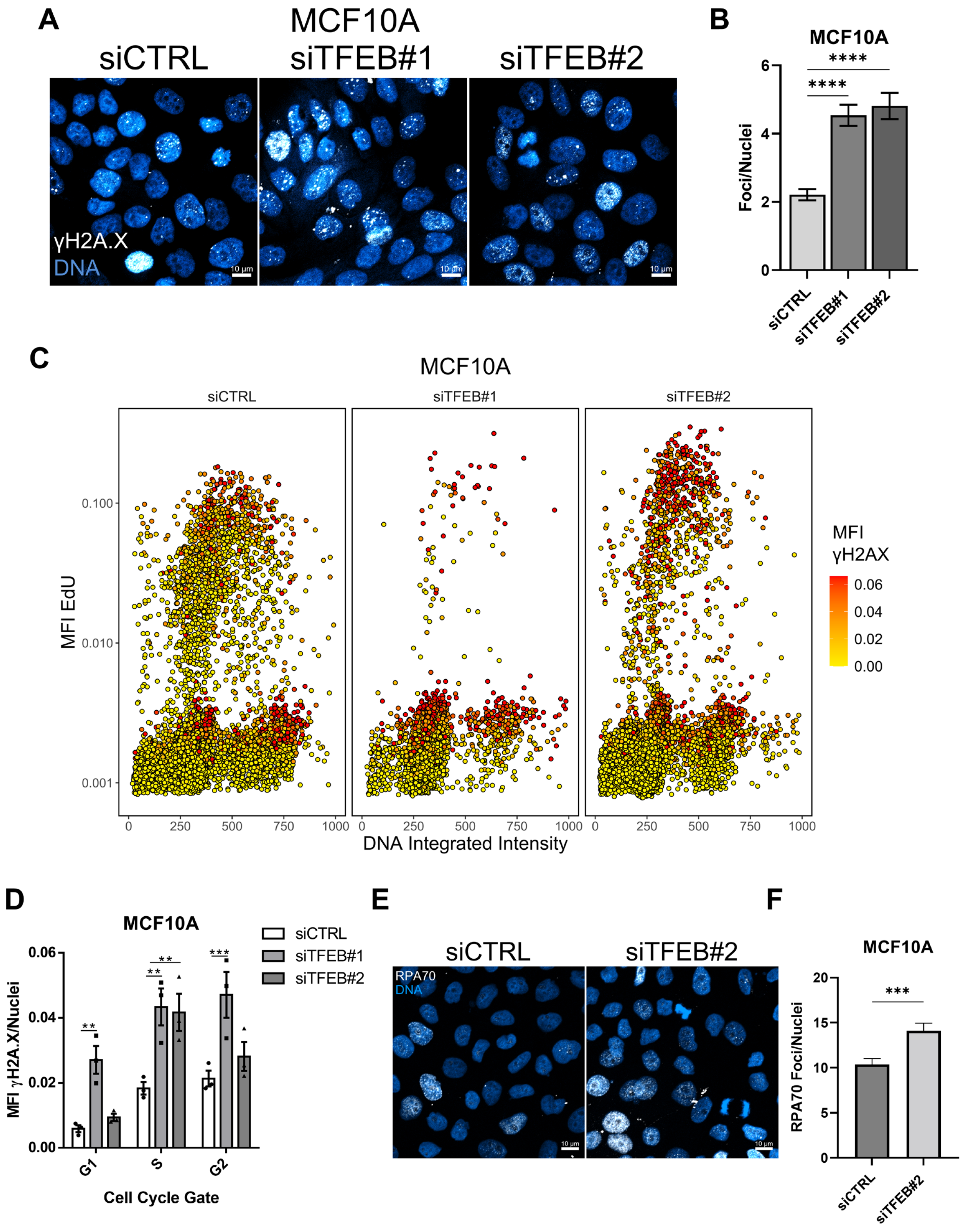

A
MCF10A
siCTRL
siTFEB#1
siTFEB#2
γH2A.X
DNA
10 µm
B
MCF10A
Foci/Nuclei
****
****
siCTRL
siTFEB#1
siTFEB#2
C
MCF10A
siCTRL
siTFEB#1
siTFEB#2
MFI EdU
0.100
0.010
0.001
DNA Integrated Intensity
0
250
500
750
1000
MFI
γH2AX
0.06
0.04
0.02
0.00
D
MCF10A
MFI γH2A.X/Nuclei
0.06
0.04
0.02
0.00
**
**
**
***
G1
S
G2
Cell Cycle Gate
siCTRL
siTFEB#1
siTFEB#2
E
siCTRL
siTFEB#2
RPA70
DNA
10 µm
F
MCF10A
RPA70 Foci/Nuclei
20
15
10
5
0
***
siCTRL
siTFEB#2

Supplementary Figure 7. TFEB knockdown causes replication stress in MCF10A cells. (A, B) Immunofluorescence and quantification of γH2A.X foci in MCF10A cells 96 hours after treatment with the indicated siRNAs, n= an average of 1232 cells from two independent experiments, scale bar = 10 μM. (C, D) Quantification of γH2A.X intensity by cell cycle phase using EdU uptake and γH2A.X immunofluorescence. (E, F) Representative images and quantification of chromatin bound RPA70 foci immunofluorescence following TFEB knockdown, n = an average of 1202 cells per group, from two independent experiments, scale bar = 10 μM. *p<0.05, **p<0.01, ***p<0.001, ****p<0.0001, one-way ANOVA, two-way ANOVA (D), or t-test (F).

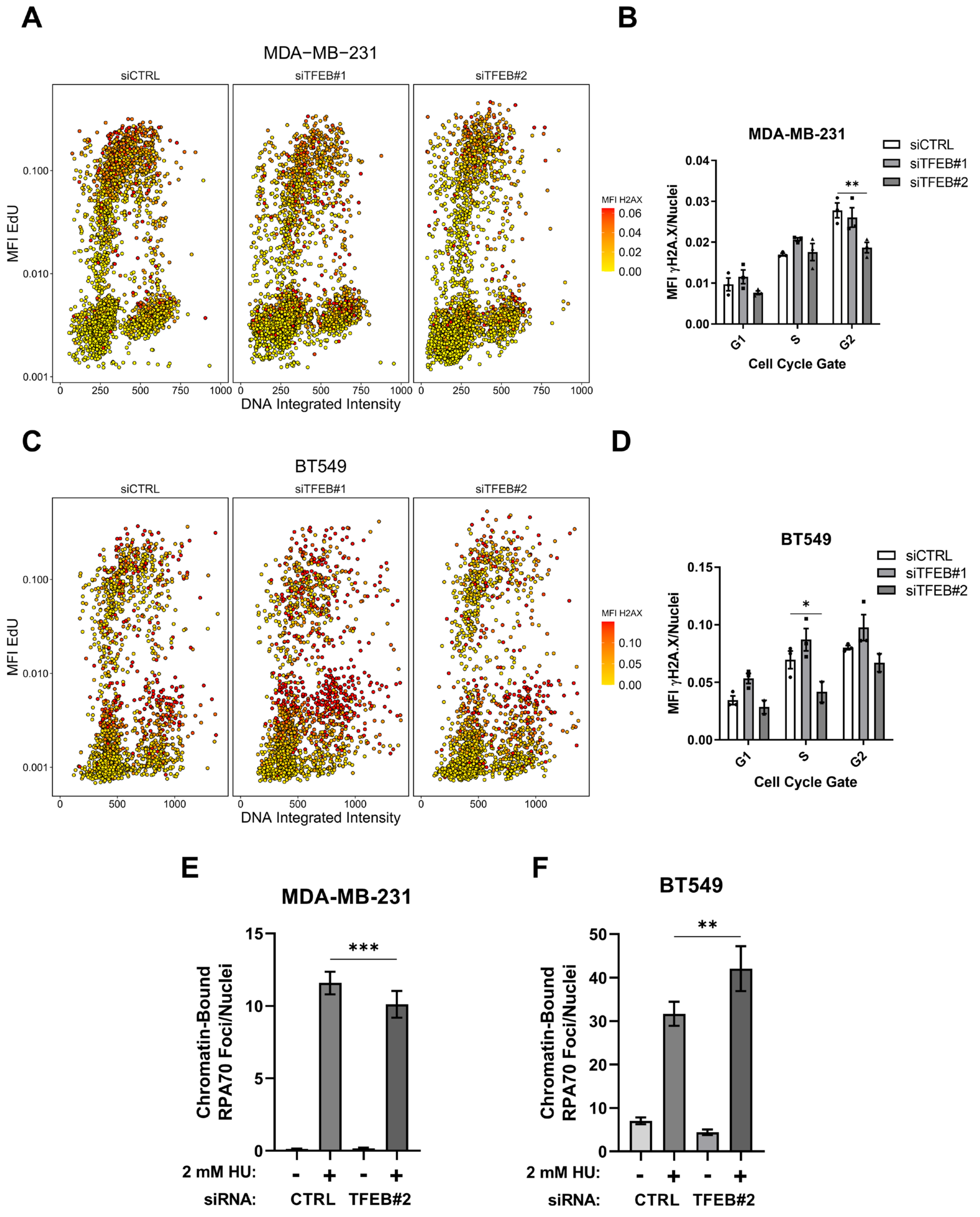
A
MDA−MB−231
siCTRL
siTFEB#1
siTFEB#2
MFI EdU
0.100
0.010
0.001
MFI H2AX
0.06
0.04
0.02
0.00
0
250
500
750
1000
DNA Integrated Intensity
B
MDA-MB-231
siCTRL
siTFEB#1
siTFEB#2
MFI γH2A.X/Nuclei
0.04
0.03
0.02
0.01
0.00
**
G1
S
G2
Cell Cycle Gate
C
BT549
siCTRL
siTFEB#1
siTFEB#2
MFI EdU
0.100
0.010
0.001
MFI H2AX
0.10
0.05
0.00
0
500
1000
DNA Integrated Intensity
D
BT549
siCTRL
siTFEB#1
siTFEB#2
MFI γH2A.X/Nuclei
0.15
0.10
0.05
0.00
*
G1
S
G2
Cell Cycle Gate
E
MDA-MB-231
Chromatin-Bound RPA70 Foci/Nuclei
15
10
5
0
***
2 mM HU: - + - +
siRNA: CTRL TFEB#2
F
BT549
Chromatin-Bound RPA70 Foci/Nuclei
50
40
30
20
10
0
**
2 mM HU: - + - +
siRNA: CTRL TFEB#2

Supplementary Figure 8. TFEB silencing does not induce replication stress in TNBC cells. (A-D) Quantification of γH2A.X intensity by cell cycle phase in the indicated cell line using EdU uptake and γH2A.X immunofluorescence. (E, F) Immunofluorescence quantification of chromatin-bound RPA70 foci in (E) MDA-MB-231, and (F) BT549 cells with or without TFEB knockdown treated with 2 mM hydroxyurea or control for 24 hours. (E) N = average of 1003 cells from two independent experiments, (F) N = average of 308 cells from two independent experiments. *$p<0.05$, **$p<0.01$, ***$p<0.001$, two-way ANOVA (B, D), Kruskal-wallis test (E, F).
